# Parity Metamaterials and Dynamic Acoustic Mimicry

**DOI:** 10.34133/research.0826

**Published:** 2025-08-13

**Authors:** Jinjie Shi, Hongchen Chu, Aurélien Merkel, Chenkai Liu, Johan Christensen, Xiaozhou Liu, Yun Lai

**Affiliations:** ^1^ MOE Key Laboratory of Modern Acoustics, National Laboratory of Solid State Microstructures, School of Physics, Collaborative Innovation Center of Advanced Microstructures, and Jiangsu Physical Science Research Center, Nanjing University, Nanjing 210093, China.; ^2^ School of Physics and Technology, Nanjing Normal University, Nanjing 210023, China.; ^3^ Université de Lorraine, CNRS, Institut Jean Lamour, F-54000 Nancy, France.; ^4^ IMDEA Materials Institute, Calle Eric Kandel, 2, Getafe, 28906 Madrid, Spain.

## Abstract

While parity transformation represents a fundamental symmetry operation in physics, its implications remain underexplored in metamaterial science. Here, we introduce a framework leveraging parity transformation to construct parity-inverted counterparts of arbitrary 3-dimensional meta-atoms, enabling the creation of parity-engineered metamaterial slabs. We demonstrate that the synergy between reciprocity and parity transformation, distinct from mirror operation, guarantees undistorted wave transmission across exceptional bandwidths, independent of structural configuration or meta-atom design specifics. Furthermore, these metamaterials exhibit dynamic acoustic mimicry capability, enabling adaptive blending of reflected signatures into surrounding environments while preserving transmitted wavefront integrity. Validated through numerical simulations and experimental prototypes, this breakthrough offers transformative potential for acoustic camouflage applications, particularly for sonar systems. Our findings reveal fundamental implications of parity transformation in artificial materials, establishing parity engineering as a paradigm for designing ultrabroadband functional materials with unprecedented operational versatility.

## Introduction

**Fig. 1.**
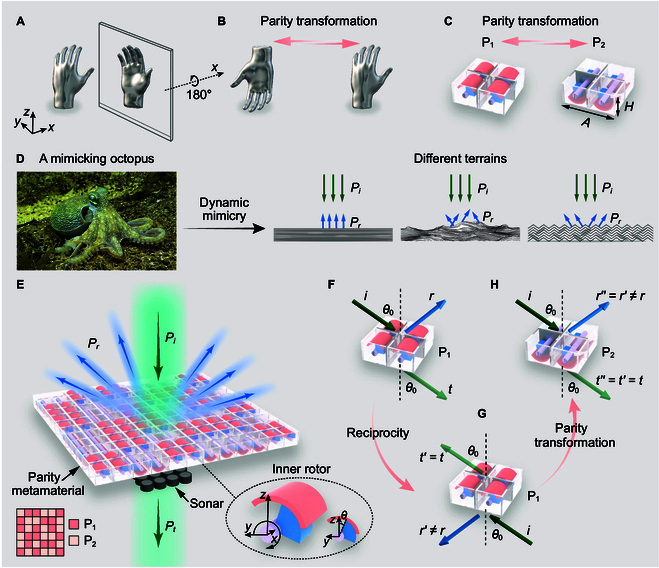
A parity metamaterial composed of a meta-atom and its parity-inverted counterpart. (A and B) The hand, its mirror image, and its parity-inverted counterpart. The mirror image and the parity-inverted counterpart are related by a 180° rotation along the *x* axis. (C) The 3D meta-atom and its parity-inverted counterpart design related by parity transformation. (D) The concept of acoustic mimicry to imitate different terrains, including a flat terrain, a rugged terrain, and a periodic terrain, like an octopus. (E) The illustration of a parity metamaterial based on a random distribution of P_1_ and P_2_. Bottom right inset provides a magnified picture of the inner rotor. (F to H) Reciprocity and parity transformation rigorously prove that the meta-atom and its parity-inverted counterpart have the same transmission but different reflection coefficients.

Parity transformation, the symmetry operation [1] involving a change of the sign in all spatial coordinates, i.e., $\left(x,\;y,\;z\right)\to \left(-x,-y,-z\right)$, has many profound impacts in physics. Applying parity transformation to an arbitrary object creates its unique counterpart as a rotation of its mirror image with reversed chirality, as exemplified by a hand and its image in the mirror in Fig. 1A and B. Recently, parity–time (PT) symmetry [2–5] has been exploited to reveal the existence of exceptional points [6–11], opening a promising field of non-Hermitian physics [12]. However, to date, there has been little discussion on the possibility of applying parity transformation alone in acoustic metamaterials [13–20] and metasurfaces [21,22], which have markedly broadened the boundaries of acoustic materials [23–42] and enabled novel functions such as acoustic cloaks and illusions [23,24,29,42] over the past decades.

Sonar domes are widely used in air and underwater environments to house and protect sonars [43,44]. To ensure accurate signal acquisition, they are typically made of effectively homogeneous materials that support broadband undistorted transmission. However, this uniformity also retains the reflection signature of the protected sonars, making it impossible to acoustically blend into surrounding environments. On the other hand, conventional digital coding metasurfaces [45–52] can alter the reflection signature, but they typically block or disrupt transmission signals, thereby disabling the functionality of the sonar. Overall, it is highly desirable to achieve dynamic acoustic camouflages [42] in reflection while keeping the sonar function intact in an ultrabroad spectrum. This requires the combination of effective homogeneity and inhomogeneity in a single material, which has been seldomly discussed.

In this work, we apply parity transformation to design a class of acoustic metamaterials denoted parity metamaterials. Parity transformation relates an arbitrary 3-dimensional (3D) meta-atom with its unique parity-inverted counterpart. They constitute a pair of building blocks (Fig. 1C) for a special metamaterial slab called parity metamaterial (Fig. 1E), which can keep transmission wavefront undistorted in an extremely broad spectrum, while dynamically tuning the wavefront in reflection to mimic those from a periodic terrain, a rugged terrain, and a flat terrain, etc. These metamaterials thus enable dynamic acoustic mimicry (Text S1 and Fig. S1) to blend into the acoustic environment. At the same time, ultrabroadband undistorted transmission is guaranteed irrespective of the dynamical mimicry, which is extremely important for sonar detection. Therefore, this metamaterial offers a way to realize dynamic acoustic camouflage for advanced sonar systems, just like octopuses that can adapt their color and form to blend into their surroundings while still perceiving the environment (Fig. 1D). Our theory unveils the principle of parity engineering in metamaterials and metasurfaces, promising a profound impact in multiple disciplines.

## Results

### Theory and design of parity metamaterials

We consider an arbitrary 3D meta-atom and its parity-inverted counterpart, as denoted by P_1_ and P_2_, respectively. To accord with the working wavelength, the basic units of the metamaterial are composed of an array of 2×2 meta-atoms P_1_ or P_2_, as depicted in Fig. 1C. The adjacent basic units are separated by hard boundaries to minimize the coupling between them. The length and thickness of the array are specified as A=60mm and H=24mm, respectively. To achieve dynamic tunability of the parity metamaterials, we designed the meta-atom and its parity-inverted counterpart to be rotatable. The magnified view of the inner rotor is displayed in the bottom-right inset of Fig. 1E. The curved plate can be rotated to tune the reflection phase, and the connection to the shaft is set to be asymmetric to remove all symmetry in the metastructure. The detailed parameters of the meta-atom can be found in Text S2 and Fig. S2. Figure 1E depicts the schematic diagram of the parity metamaterial slab composed of a selected arrangement of the meta-atom and its parity-inverted counterpart, which can tune the reflection while keeping transmission unaffected, if the system is reciprocal.

The underlying physical principle is described as follows. We first consider the scattering properties of this pair of metastructures (P_1_ and P_2_), assuming that the transmission and reflection coefficients of P_1_ in Fig. 1F are denoted as *t* and *r*, respectively. When the system is reciprocal, the reciprocity theorem [53–55] asserts that the exchange of the incidence and transmission channels does not change the transmission coefficient, i.e., $t^{\prime }=t$, as shown in Fig. 1G. On the contrary, the reflection coefficient can differ markedly after the interchange, i.e., $r^{\prime }\neq r$. Subsequently, we apply a parity operation to the system, as shown in Fig. 1H. P_1_ is transformed into its counterpart, i.e., P_2_, which has the same transmission coefficient as P_1_, i.e., $t^{\prime \prime }=t^{\prime }=t$. This equivalence is regardless of the details of the metastructures, as well as the frequency and the incident angle. In reflection, contrarily, we have $r^{\prime \prime }=r^{\prime }\neq r$; thus, there is a phase difference between *r′* and *r* over a wide spectrum, which can modulate the reflection from this metamaterial. Moreover, the phenomena here can also be explained by a theory based on the transfer matrix method in stratified media (Text S3 and Fig. S3).

### Ultrabroadband and wide-angle undistorted transmission

**Fig. 2.**
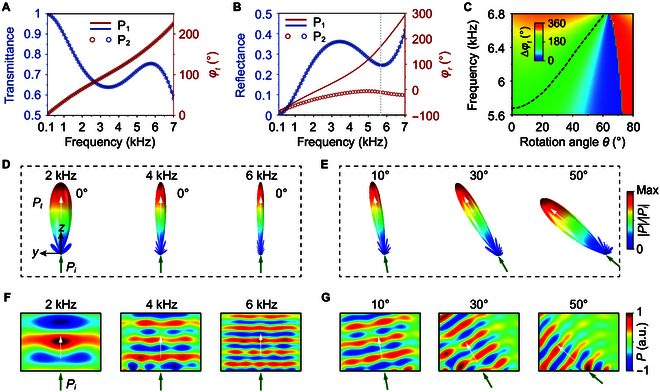
Transmission and reflection properties of P_1_ and P_2_. (A) Transmittance and transmission phase spectra of P_1_ and P_2_. (B) Reflectance and reflection phase spectra of P_1_ and P_2_. (C) Reflection phase difference between P_1_ and P_2_ as a function of the rotation angle and frequency. (D) Simulated 3D far-field radiation power patterns in transmission under normal incidence at 2, 4, and 6 kHz. (E) Simulated 3D far-field radiation power patterns in transmission under incident angles of 10°, 30°, and 50° at 4 kHz. Each subpanel in (D) and (E) is normalized. (F to G) Simulated near-field distributions of the transmitted acoustic field in the *yz* plane. Here, the colors indicate the normalized pressure of the acoustic field. a.u., arbitrary units.

In the following, we present the transmission and reflection properties of P_1_ and P_2_. Figure 2A and B illustrates, respectively, the calculated transmittance and transmission phase, as well as the reflectance and reflection phase of P_1_ and P_2_, as functions of the frequency. The direction of the incident wave is along the *z* direction. Here, acoustic dissipation is neglected for simplicity, but this principle also applies to general dissipated systems. From Fig. 2A, it is seen that the transmittance and transmission phase of P_1_ and P_2_ are identical over an ultrabroad spectrum ranging from 0.1 to 7 kHz (Text S4 and Fig. S4). The reflectance is also the same, but there is a substantial difference in the reflection phase, as depicted in Fig. 2B. In particular, the phase difference, Δ*φ_r_*, reaches the maximum value of 180° at a frequency of 5.68 kHz (denoted by a gray vertical line). By rotating P_1_ and P_2_ simultaneously, the phase difference in reflection can be conveniently tuned. Figure 2C illustrates the reflection phase difference between P_1_ and P_2_, i.e., $∆\varphi_{r}$, as a function of the rotation angle of the rotor and frequency, which covers the whole range of 360° around 5.68 kHz. The black dashed line represents the condition of $∆\varphi_{r}=180{}^{\circ}$. We emphasize that rotation does not change the condition of equal transmittance and $∆\varphi_{t}=0{}^{\circ}$ over the whole spectrum from 0.1 to 7 kHz (Text S5 and Fig. S5). This condition guarantees that the transmitted acoustic wavefront is the same as that of the incidence in an ultrabroad spectrum, independent of the arrangement of P_1_ and P_2_.

To demonstrate this unique feature, we consider a parity metamaterial composed of randomly distributed P_1_ and P_2_ illuminated by an incident plane wave of different frequencies and incident angles. The arrangement of P_1_ and P_2_ is shown in the bottom-left inset of Fig. 1E. By using finite-element software COMSOL Multiphysics, the 3D far-field radiation power patterns in transmission are calculated under normal incidence at 2, 4, and 6 kHz, as shown in Fig. 2D. The direction of transmission (*P_t_*) is the same as that of the incidence (*P_i_*) for all frequencies. In Fig. 2E, we plot the 3D far-field radiation power patterns in transmission for incident angles of 10°, 30°, and 50° at 4 kHz. It is observed that the direction of transmission (*P_t_*) aligns consistently with the direction of incidence (*P_i_*) for all angles. These effects are further verified by the calculated near-field distributions, as shown in Fig. 2F and G, respectively. In other words, such a disordered metamaterial functions like an ordered metamaterial in transmission, ensuring its feasibility in important applications such as acoustic detection and sonar technology [43,44,56].

### Parity transformation versus mirror operation

**Fig. 3.**
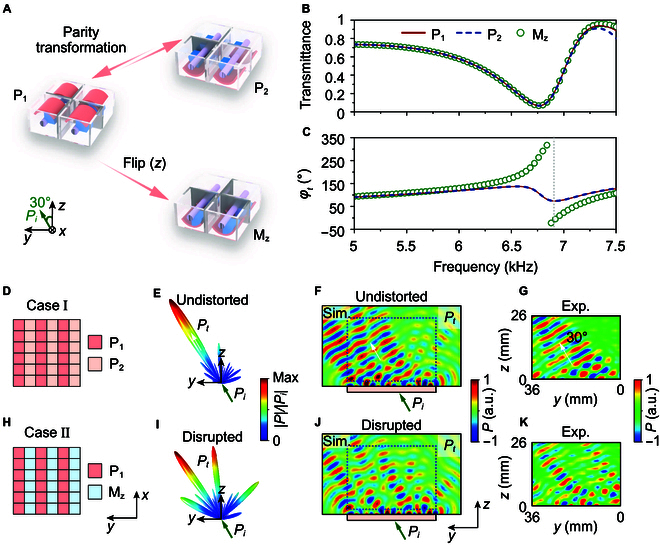
Comparison between parity transformation and mirror operation. (A) Schematic diagrams of P_1_, P_2_, and M_z_. M_z_ is the mirror image of P_1_ along the *z* direction. (B and C) Transmittance and transmission phase spectra of P_1_, P_2_, and M_z_ under the illumination of an incident angle of 30°. (D and H) Metamaterial design for cases I and II, respectively. (E and I) Simulated 3D far-field radiation power patterns in transmission for cases I and II, respectively. (F, J, G, and K) Simulated (F and J) and measured (G and K) near-field distributions of the transmitted field for case I (F and G) and case II (J and K) in the *yz* plane.

To elucidate the essential role of parity transformation, we compare it with the mirror operation, i.e., upside-down flip, namely, $\left(x,\;y,\;z\right)\to \left(x,\;y,-z\right)$. When the metastructure exhibits a $C_{4}^{v}$ symmetry in the *xy* plane, there is no difference between $\left(x,\;y,\;z\right)\to \left(-x,-y,-z\right)$ and $\left(x,\;y,\;z\right)\to \left(x,\;y,-z\right)$ [57,58]. However, the difference becomes huge when the metastructure has no symmetry, which is the case here. Figure 3A depicts the schematic diagrams of P_1_, P_2_, and the structure generated by mirror operation, M_z_, respectively. It is seen that M_z_ is different from P_2_. In Fig. 3B and C, we plot the transmittance and transmission phase spectra of P_1_, P_2_, and M_z_ under the illumination of an incident angle of 30°, respectively, as shown by the green arrow in Fig. 3A. P_1_ and P_2_ have the same transmittance and transmission phase, as strictly protected by reciprocity and parity transformation. However, M_z_ and P_1_ exhibit distinctly different transmission phase around 6.9 kHz. A comprehensive discussion is available in Text S6 and Fig. S6. The wide-angle transmission behaviors of P_1_, P_2_, and M_z_ are shown in Text S7 and Fig. S7.

Such a fundamental difference between parity transformation and mirror operation is further experimentally verified. We construct 2 metamaterials with the sequences P_1_P_2_P_1_P_2_P_1_P_2_ (case I) and P_1_M_z_P_1_M_z_P_1_M_z_ (case II), as illustrated in Fig. 3D and H, respectively. The simulated 3D far-field radiation power patterns in transmission for cases I and II under an incident angle of 30° at 6.9 kHz are shown in Fig. 3E and I, respectively. The transmitted wave (*P_t_*) is undistorted in Fig. 3E but disrupted in Fig. 3I. Such a distinct difference originates in the transmission phase difference between P_1_ (P_2_) and M_z_, which reaches 87° at 6.9 kHz (denoted by a gray vertical dotted line in Fig. 3C). This huge difference is also observed in the calculated and measured near-field distributions for cases I and II, as shown in Fig. 3F, G, J, and K, respectively. The measured regions are marked by the blue dotted boxes in the *yz* plane, as shown in Fig. 3F and J. The experimental and numerical results approximately agree with each other, both confirming that it is the parity transformation instead of the mirror operation that can preserve the transmission wavefront.

### Dynamic acoustic mimicry

**Fig. 4.**
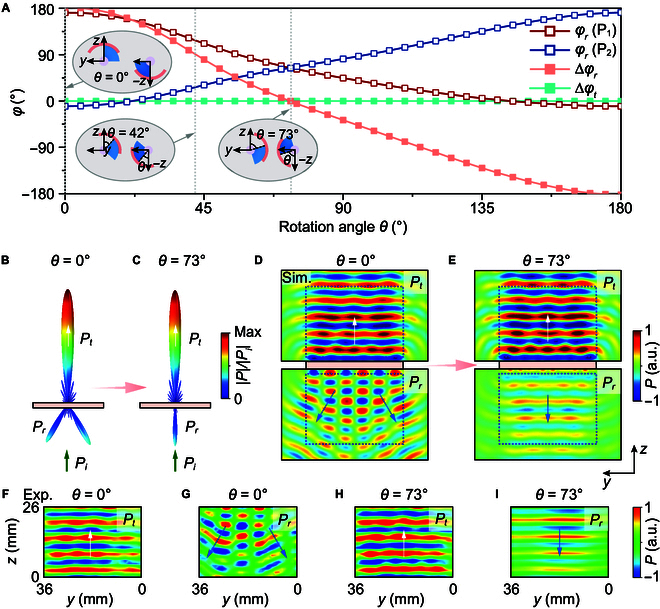
Dynamic acoustic mimicry using a parity metamaterial. (A) Reflection phase spectra of P_1_ and P_2_ and their difference in reflection and transmission as functions of the rotation angle *θ* at 5.68 kHz. Insets show the magnified views of the inner rotors of P_1_ and P_2_. (B and C) Simulated 3D far-field radiation power patterns under normal incidence at 5.68 kHz for θ=0° and θ=73°, respectively. (D and E) Simulated corresponding near-field distributions in the *yz* plane. (F to I) Measured acoustic field distributions of transmitted (F and H) and reflected (G and I) waves under normal incidence at 5.68 kHz when θ=0° (F and G) and θ=73° (H and I).

In the following, we numerically and experimentally demonstrate the realization of dynamic acoustic mimicry. By rotating the rotors of the designed parity metamaterial, it is possible to alter the reflection to emulate that from a periodic terrain, a rugged terrain, and a flat terrain, while keeping the transmission wavefront undistorted (Text S8 and Fig. S8). The insets of Fig. 4A portray the magnified views of the inner rotors of P_1_ and P_2_, wherein the rotors of both P_1_ and P_2_ are simultaneously rotated by the same angle, such that P_1_ and P_2_ can always be transformed into each other via parity transformation. Consequently, P_1_ and P_2_ always have identical transmittance (Fig. S9) and transmission phase (Fig. 4A), regardless of the rotation angle. On the other hand, the reflection phase difference between P_1_ and P_2_ varies markedly with the rotation angle. Figure 4A depicts the calculated reflection phase of P_1_ and P_2_, as well as their difference as functions of the rotation angle *θ* under normal incidence at a working frequency of 5.68 kHz. The reflection phase difference $∆\varphi_{r}$ varies from −180° to 180°, while the transmission phase difference $∆\varphi_{t}$ remains 0°. This indicates that the rotation angle *θ* offers a freedom to dynamically tune the reflection without changing the transmission wavefront.

Here, we demonstrate the switching of reflection from 2-beam reflection to specular reflection, while maintaining undistorted transmission all the time. The parity metamaterial is the same as that shown in Fig. 3D. Figure 4B and C shows the simulated 3D far-field radiation power patterns under normal incidence at 5.68 kHz for θ=0° and θ=73° (denoted by gray vertical dotted lines in Fig. 4A), respectively. The direction of transmission (*P_t_*) is the same as that of the incidence (*P_i_*) for both cases. On the other hand, the reflection changes from the case of splitting into 2 beams (for θ=0°) to the case of specular reflection (θ=73°). This is because Δ*φ_r_* changes from 180° to 0° when *θ* changes from 0° to 73°. The switching phenomenon is further verified by simulated and measured near-field distributions, which are shown in Fig. 4D to I, respectively. The scanned areas correspond to the blue dotted boxes in Fig. 4D and E. The measured results coincide excellently with the numerical results, confirming that the transmitted wave (*P_t_*) maintains a plane wavefront for the 2 cases. For reflection, the angle of reflection is $\theta_{r}=30.2{}^{\circ}$ $\left\{\theta_{r}={\text{sin}}^{-1}\left[\lambda /\left(2A\right)\right]\;\right\}$ (Text S9) at a rotation angle of θ=0°, as shown by the green arrows in Fig. 4D. The quantitative analysis of the transmission and reflection behaviors is shown in Text S10 and Fig. S11. In addition, the case for θ=42° is shown in Text S8 and Fig. S10, displaying the simultaneous existence of 3-beam reflection and undistorted transmission. Therefore, dynamic acoustic mimicry can be realized using the rotation angle *θ* as an extra degree of freedom in the metastructure design.

We also demonstrated the cases of diffuse reflection and reflection holography (Texts S11 and S12 and Figs. S12 and S13). The results again confirm the conclusion: The acoustic reflection signatures can be freely altered to mimic the sophisticated acoustic environment, while keeping the transmitted wavefront unchanged in an ultrabroad spectrum, which is crucial for sonar. This functionality cannot be achieved by conventional digital coding metasurfaces [45–52] (Text S13 and Fig. S14).

### Temporal acoustic camouflage

**Fig. 5.**
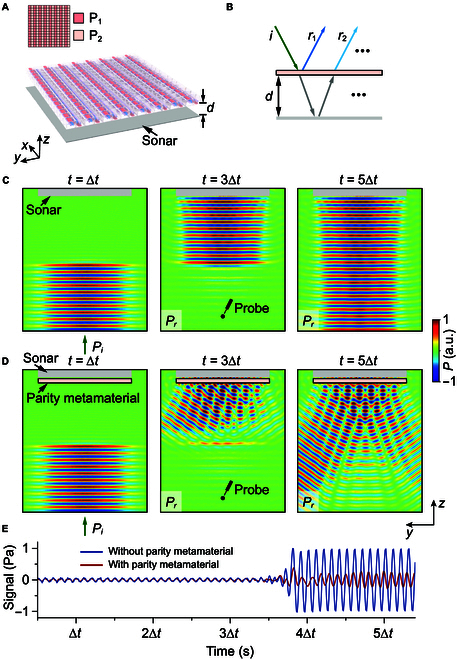
Temporal acoustic camouflage via a sonar integrated with a parity metamaterial. (A) Schematic diagram of a sonar integrated with a parity metamaterial. Inset shows the design of the parity metamaterial. (B) The mechanism of the acoustic camouflage. (C and D) Snapshots of a Gaussian pulse incident on a sonar without (C) and with (D) a parity metamaterial, where ∆t=1ms. The left panels of (C) and (D) show the incident pulse, while the middle and right panels display the reflected signals. (E) Simulated reflection signals received at the probe, both without and with a parity metamaterial.

The integration of parity metamaterials with sonar systems offers the potential for temporal camouflage. This effect is vividly demonstrated by comparing the reflection signals of a Gaussian pulse incident on a sonar, both without and with a parity metamaterial. The pulse is a time-domain Gaussian signal with a duration of 1 s and a center frequency of 10 kHz. In accordance with the working wavelength, the basic units of the parity metamaterial are composed of only one meta-atom P_1_ or its parity-inverted counterpart P_2_, as shown in Fig. 5A. In our simulations, the sonar is modeled as an impedance boundary with an impedance of 38Z_0_, where Z_0_ is the impedance of air. Under this condition, the simulated sonar provides approximately 90% reflection.

The parity metamaterial is closely attached to the sonar (just like the necessary sonar domes in practical sonar systems). In this case, the reflections from the metamaterial and the sonar itself both influence the overall reflection. By meticulously adjusting the rotation angle *θ* of the rotors in the parity metamaterial, as well as the distance *d* between the metamaterial and the sonar, it is possible to achieve destructive interference between the reflections from the sonar and those from the parity metamaterial. This results in a substantial reduction of the overall signal. The mechanism behind the acoustic camouflage is illustrated in Fig. 5B. The total reflection *R* can be expressed as

$$\begin{array}{c} R=r_{1}+\sum_{n=1}^{\infty }t_{1}^{2}{r_{1}^{\prime }}^{n-1}r_{2}^{n}e^{-2\text{in}\varphi_{d}}=r_{1}+t_{1}^{2}r_{2}e^{-2i\varphi_{d}}{\left(1-r_{1}^{\prime }r_{2}e^{-2i\varphi_{d}}\right)}^{-1} \end{array}$$ (1)

where *r*_1_ and $r_{1}^{\prime }$ denote the reflection coefficients on the front and back interfaces of the parity metamaterial, *r*_2_ is the reflection coefficient of the sonar, *t*_1_ is the transmission coefficient of the parity metamaterial, and $\varphi_{d}$ indicates the phase change through the air layer. By setting R=0, the overall specular signal can be eliminated. Under this condition, we only need to consider the 0th-order transmission and reflection coefficients of the parity metamaterial and sonar. The phase shift can be expressed as $\varphi_{d}=\mathrm{kd}$, where *k* is the wave number. For example, when the rotation angle of the rotors is θ=75°, the distance between the metamaterial and the sonar is calculated as d=5.9mm. The details are shown in Text S14 and Fig. S15.

Figure 5C shows the snapshots of the incident and reflected Gaussian pulse without the parity metamaterial. As expected, a strong specular reflection is observed when the pulse directly impinges on the sonar. In contrast, when the parity metamaterial covers the sonar, the specular reflection is substantially reduced, and the scatterings in other directions emerge, as shown in Fig. 5D. To quantify the temporal camouflage effect, we simulated the reflected signals received at a probe located at a certain distance from the sonar. The results, shown in Fig. 5E, demonstrate a marked reduction in the reflected signal intensity when the parity metamaterial is present. Specifically, the reflected signal strength is reduced to below 7% of its original value, and this value is expected to decrease further at greater distances. This effect makes the sonar system more difficult to be detected. Simultaneously, the parity metamaterial preserves the undistorted ultrabroadband transmission, ensuring the effectiveness of sonar detection.

## Conclusion

We would like to emphasize that the concept of parity metamaterials is fundamentally different from PT-symmetric metamaterials. PT-symmetric metamaterials [2–5] are non-Hermitian systems with global PT symmetry, while the parity metamaterials do not require any global symmetry at all. On the other hand, PT-symmetric systems require delicately balanced loss and gain, while the functionalities of parity metamaterials are inherently robust to loss (Text S15 and Fig. S16) because both the reciprocity principle and parity transformation are uninfluenced by loss.

Parity metamaterials are fundamentally different from previously reported metamaterials studied in topological acoustics [59–63]. In topological systems, the wave behavior is governed by the bulk band structure, which arises from the periodic arrangement of a single type of unit cell with carefully designed internal symmetry and intercell coupling. These systems often exhibit edge-localized states, valley vortex fields, or other nontrivial band topology effects. In contrast, parity metamaterials proposed here are constructed using a pair of meta-atoms, i.e., a meta-atom and its unique parity-inverted counterpart. Our design is not constrained by lattice periodicity and enables aperiodic arrangements, through which the reflection signature can be tailored. This mechanism unlocks a new class of wavefront control, wherein broadband undistorted transmission is guaranteed irrespective of tunable reflection shaping, enabling applications such as camouflaged sonar domes.

On the other hand, asymmetry has been recently adopted to achieve exceptional points for unidirectional absorption [64]. Here, asymmetry is applied in the design of meta-atoms for dynamic acoustic mimicry. The approach of parity metamaterials generally applies to meta-atoms of any symmetry. Compared with symmetric meta-atoms, asymmetric meta-atoms can provide many more degrees of freedom for tuning the reflection, thereby enabling dynamic acoustic mimicry.

We should note that parity metamaterials reveal the fundamental difference between parity transformation $\left(x,\;y,\;z\right)\to$$\left(-x,-y,-z\right)$ and mirror operation $\left(x,\;y,\;z\right)\to \left(x,\;y,-z\right)$, which was applied in previous optical metasurface designs [57,58]. This fundamental difference is manifested by the important advantage that parity metamaterials can be constructed from arbitrary building blocks, including ones with chirality and no symmetry at all, which is a huge advance in contrast to the previous optical designs. This difference also releases a large amount of new freedom that enables the dynamical acoustic mimicry, which can flexibly simulate the acoustic signatures of a periodic terrain, a rugged terrain, and a flat terrain, etc. Furthermore, the acoustic camouflage performance is also demonstrated in temporal domains.

In summary, we introduce the fundamental symmetry operation, parity transformation, to design a new class of metamaterials denoted as parity metamaterials. These metamaterials are constituted by an arbitrary 3D meta-atom and its unique parity-inverted counterpart. The combination of parity transformation and reciprocity allows for dynamic acoustic mimicry in reflection without distorting the transmitted wavefront across a wide spectrum. This approach is universal and applies to all types of structures and materials, as long as they are reciprocal. The constituent meta-atoms do not necessitate any specific symmetry and work robustly with loss. Although the demonstration here is conducted using airborne sound, it can also be extended to underwater scenarios (Text S16 and Fig. S17). It is also effective when the thickness of the metamaterial is much larger (Text S17 and Fig. S18). Our findings uncover the profound role of parity transformation in ultrabroadband wave manipulation, establishing parity engineering as a transformative paradigm for artificial material design, with implications spanning acoustic camouflage, adaptive metasurfaces, and next-generation communication systems.

## Methods

### Numerical simulations

The full-wave simulations are performed using the commercial finite element software COMSOL Multiphysics. In the calculations, the parameters of air are set as $\rho_{0}=1.21\;\mathrm{kg}/m^{3}$ and $c_{0}=343\;m/s$. The impedance of air is $Z_{0}=\rho_{0}c_{0}=415.03\;\mathrm{Pa}\cdot s/m^{3}$. The structures are fabricated using photopolymer resin, with a mass density $\rho =1,300\;\mathrm{kg}/m^{3}$ and speed of sound c=716m/s. Acoustic impedance is $Z=\rho c\approx 9.3\times 10^{5}$ Pa·s/m. Given that the acoustic impedance of air is approximately 415 Pa·s/m, this results in an impedance contrast exceeding 2,243×, which justifies the treatment of the resin as acoustically rigid. The background pressure field is used in Figs. 2 to 4. The periodic boundary condition is set in the *x* and *y* directions, and perfectly matched layers are adopted in the *z* direction to reduce the reflection. In Fig. 5, a Gaussian wave is utilized.

### Experimental measurements

All samples are fabricated with resin using stereolithography 3D printing techniques (0.2 mm in precision). All the rotors are printed separately to facilitate rotational operations during the experiment. The rotor angles are manually adjusted using a standard protractor (angle ruler). Before each measurement, we align the rotor by visually matching the angle between the curved plate and a reference line with the aid of a handheld protractor. While this method is simple, it allows us to control the rotor angle with an estimated accuracy of ±2° to 3°. The whole size of the sample is 360mm×360mm×360mm.

The experimental configuration is depicted in Fig. S19. To generate a quasi-plane wave, a speaker array (Five HiVi B1S) equipped with a parabolic mirror is meticulously constructed. A microphone (GRAS 46BE) is affixed to a movable stage to systematically scan the distribution of the acoustic field with a step size of 10 mm. To mitigate the influence of waves propagating around the specimen, sound-absorbing foams are affixed in its vicinity. The 2 measured regions on the sides of reflection and transmission in the *xz*-plane are shown as 2 blue rectangular areas in Fig. S19A. Both regions are 36mm×26mm in size and are positioned 2 cm away from the sample. The photo of the experimental setup is shown in Fig. S19B. The measured reflected field distribution is obtained by subtracting the incident field (measured without the sample) from the total field (measured with the sample). The experiment is carried out in an anechoic room to minimize reflection and noise.

## Data Availability

The data that support the plots within this paper and other findings of this study are available from the corresponding author upon reasonable request.

## Supplementary Materials

Supplementary 1 Texts S1 to S17 Figs. S1 to S19
