# Supplementary material for: Parity Metamaterials and Dynamic Acoustic Mimicry
Source: Research (Wash D C). 2025 Aug 13;8:0826. doi: 10.34133/research.0826 (PMC12349962; doi:10.34133/research.0826)
Supplement: Supplementary 1 — Texts S1 to S17 Figs. S1 to S19 [file research.0826.f1.docx]

Supplementary Materials

Parity Metamaterials and Dynamic Acoustic Mimicry

Jinjie Shi^1^†, Hongchen Chu^2^†, Aurélien Merkel^4^, Chenkai Liu^1^, Johan Christensen^3*^, Xiaozhou Liu^1*^, and Yun Lai^1*^

^1^MOE Key Laboratory of Modern Acoustics, National Laboratory of Solid State Microstructures, School of Physics, Collaborative Innovation Center of Advanced Microstructures, and Jiangsu Physical Science Research Center, Nanjing University, Nanjing 210093, China.

^2^School of Physics and Technology, Nanjing Normal University, Nanjing 210023, China.

^3^IMDEA Materials Institute, Calle Eric Kandel, 2, 28906, Getafe, Madrid, Spain.

^4^Université de Lorraine, CNRS, Institut Jean Lamour, F-54000 Nancy, France.

^*^Address correspondence to: Johan Christensen; [johan.christensen@imdea.org](mailto:johan.christensen@imdea.org), Xiaozhou Liu; [xzliu@nju.edu.cn](mailto:xzliu@nju.edu.cn) and Yun Lai; laiyun@nju.edu.cn

†These authors contributed equally to this work

**This file includes:**

Texts: S1 to S17

Figs: S1 to S19

**Supplementary Texts**

Text S1. Camouflaged sonar dome based on parity metamaterials

Sonar domes are commonly employed alongside sonar systems and are widely utilized in underwater vessels. Their primary purpose is to house and protect electronic equipment used for detection, navigation, and ranging. To ensure the effectiveness of sonar detection, sonar domes must possess ultra-broadband undistorted acoustic transmission, which is satisfied by the unique function of the parity metamaterials proposed here. Interestingly, the dynamic acoustic mimicry of parity metamaterials is capable of changing the signature of sonar in reflection. When the sonar dome introduces diffuse reflection (depicted by blue color) or other signatures, while preserving the undistorted ultra-broadband transmission (depicted by red color), as shown in Fig. S1, the signature of sonar in reflection is changed, making the sonar system more difficult to be detected by other sonars.

Text S2. Detailed parameters of the meta-atom

The diagram of P_1_ is shown in Fig. S2, which is the $2\times2$ array of the meta-atom. The length and thickness of the meta-atom are $a=30 mm$ and $H=24 mm$, respectively. Particularly, the meta-atom is ingeniously designed to be a rotatable rotor. For clarity, Figure S2 shows the front view, right view, and top view of the meta-atom. The curved plate (the pink part) has a span of $\theta_{2}=120^{\circ}$ whereby the inner and outer radius of the curved plate are $R_{1}=10 mm$ and $R_{2}=12 mm$, respectively. The depth of the curved plate is $d_{2}=22 mm$. The fan structure (the blue part) has a span of $\theta_{3}=90^{\circ}$ and the thickness is $d_{1}=11 mm$. The middle shaft (the purple part) exhibits a radius of $r=3 mm$. The thickness of all the hard walls is $t=2 mm$. Here, the curved plate is primarily used to reflect sound waves, and it can adjust the reflection phase during rotation. The depth can also be changed to tune the transmittance. The shaft provides the meta-atom (its parity-inverted counterpart) with rotational freedom. The fan structure connects the curved plate and the shaft, and is set to be asymmetric to remove all symmetry of the meta-atom and its parity-inverted counterpart.

Text S3. Reciprocal transmission through stratified media and the parity-inverted configuration

Reciprocity of transmission in stratified media implies that, for the configuration shown in Fig. S3B, which is the reciprocal of Fig. S3A, the transmission coefficient *tʹ* is equal to *t*. The stratified structures in Fig. S3A and B are physically identical, but the incident wave in Fig. S3B is from the opposite direction of the transmitted wave in Fig. S3A**.** Despite reciprocity-protected transmission, the reflection coefficients in these two cases are not necessarily equal. In non-absorbing media, due to the unitarity condition and transmission symmetry, any difference in reflection arises solely from a phase difference.

Here, we study the transmission and reflection properties of stratified media and verify the reciprocity of transmission by using the transfer matrix method. We consider stratified media embedded in air. The upper and lower layers are characterized by effective mass densities *ρ_1_*, *ρ_2_*, bulk moduli *κ_1_*, *κ_2_*​, and thicknesses *d_1_*, *d_2_*, respectively. For an incident wave at an angle $\theta_{0}$, the transverse wave vector component $k_{x}=k_{0}sin\theta_{0}$ remains conserved at each interface. The longitudinal wave number and angle-dependent effective acoustic impedance in the n-th layer are given by:

$k_{z,n}=\sqrt{k_{n}^{2}-k_{x}^{2}},\mathrm{with}{k_{n}=\frac{\omega}{c_{n}}=\omega\sqrt{\frac{\rho_{n}}{\kappa_{n}}}, Z}_{n}^{\theta}=\frac{\rho_{n}\omega}{k_{z,n}}=\frac{Z_{n}}{cos\theta_{n}}$ (S1)

where $Z_{n}=\rho_{n}c_{n}$, and $\theta_{n}$ is the refracted angle in layer *n*, determined by Snell's law. The characteristic matrix connects the complex amplitudes of the acoustic fields across the layers as follows:

$(\begin{matrix} p_{i} \\ p_{r} \end{matrix})=M(\begin{matrix} p_{t} \\ 0 \end{matrix})$ (S2)

where the subscript *i*, *r*, and *t* represent incident, reflected, and transmitted waves, respectively.

The transfer matrix of each layer is:

$M_{n}=\left[ \begin{matrix} cos(k_{z, n}d_{n}) & iZ_{n}^{\theta}sin(k_{z, n}d_{n}) \\ i\frac{sin(k_{z, n}d_{n})}{Z_{n}^{\theta}} & cos(k_{z, n}d_{n}) \end{matrix} \right]$ (S3)

For top-side incidence (Layer 1 first), the total transfer matrix is $M_{up}=M_{1}\cdot M_{2}$​; for bottom-side incidence (Layer 2 first), it becomes $M_{down}=M_{2}\cdot M_{1}$. The transmission coefficients in Fig. S3A and B is then given by:

$t=\frac{2Z_{0}^{\theta}}{M_{up}\left[ 11 \right]Z_{0}^{\theta}+M_{up}\left[ 12 \right]+M_{up}\left[ 21 \right]{(Z_{0}^{\theta})}^{2}+M_{up}\left[ 22 \right]Z_{0}^{\theta}}$ (S4)

$tʹ=\frac{2Z_{0}^{\theta}}{M_{down}\left[ 11 \right]Z_{0}^{\theta}+M_{down}\left[ 12 \right]+M_{up}\left[ 21 \right]{(Z_{0}^{\theta})}^{2}+M_{down}\left[ 22 \right]Z_{0}^{\theta}}$ (S5)

The reflection coefficients are expressed as:

$r=\frac{(M_{up}\left[ 11 \right]Z_{0}^{\theta}+M_{up}\left[ 12 \right])-(M_{up}\left[ 21 \right]\left( Z_{0}^{\theta} \right)^{2}+M_{up}\left[ 22 \right]Z_{0}^{\theta})}{M_{up}\left[ 11 \right]Z_{0}^{\theta}+M_{up}\left[ 12 \right]+M_{up}\left[ 21 \right]\left( Z_{0}^{\theta} \right)^{2}+M_{up}\left[ 22 \right]Z_{0}^{\theta}}$ (S6)

$rʹ=\frac{(M_{\mathrm{down}}\left[ 11 \right]Z_{0}^{\theta}+M_{\mathrm{down}}\left[ 12 \right])-(M_{\mathrm{down}}\left[ 21 \right]\left( Z_{0}^{\theta} \right)^{2}+M_{\mathrm{down}}\left[ 22 \right]Z_{0}^{\theta})}{M_{\mathrm{down}}\left[ 11 \right]Z_{0}^{\theta}+M_{\mathrm{down}}\left[ 12 \right]+M_{\mathrm{down}}\left[ 21 \right]\left( Z_{0}^{\theta} \right)^{2}+M_{\mathrm{down}}\left[ 22 \right]Z_{0}^{\theta}}$ (S7)

By inserting Eq. S3 into Eqs. S4 and S5, we get $t=tʹ$. This identical relation is protected by reciprocity. While the reflection coefficients for cases in Fig. S3A and B can be different due to the asymmetry in impedance discontinuities encountered at the incident side.

In our parity-transformed metamaterial design, each pair of meta-atoms consists of a meta-atom and its parity-inverted counterpart. Figure S3C illustrates the flipped stack under the same incidence conditions as those in Fig. S3A. One may find that under a parity operation, the flipped case in Fig. S3C is identical to the reciprocal case in Fig. S3B, and the transmissions in both cases are equal, i.e., $tʹʹ=tʹ$. Since $t=tʹ$, we get $tʹʹ=t$, that is, transmission in the flipped case is equal to that in the original case. While the reflection in the flipped case $rʹʹ$ can be different from that of the original case *r*.

Text S4. Discussion on the strength of sonar signal acquisition

The strength of the signals collected by the sonar is primarily related to two factors. On one hand, the transmittance of P_1_ (P_2_) can be adjusted by the depth of the curved plate, i.e., *d_2_*. Figure S4 shows the transmittance of P_1_ (P_2_) as a function of the depth of the curved plate at the condition of $\Delta\varphi_{r}=180^{\circ}$. Clearly, the transmittance can be flexibly tuned by changing the depth of the curved plate and can even exceed 95%. On the other hand, the sonar's acquisition intensity can also be enhanced by increasing its sensitivity.

Text S5. Transmission phase difference between P_1_ and P_2_

Figure S5 shows the calculated transmission phase difference between P_1_ and P_2_ as a function of the rotation angle of the rotor and frequency, demonstrating that the transmission phase difference between P_1_ and P_2_ is zero over an exceptionally broad spectrum spanning from 0.1 to 7 kHz and across wide angles from 0° to 180°.

Text S6. Simulated sound pressure distributions of P_1_ and M_z_

To demonstrate the difference in transmission between P_1_ and M_z_, the simulated total sound pressure distributions of P_1_ and M_z_ are shown in Fig. S6. The background pressure fields with incident angles of 30° at 6.9 kHz are set below. The top and bottom are set as perfectly matched layers, where the red lines represent periodic boundary conditions. Owing to inherent structural disparities, the excitation modes inherently diverge, ultimately yielding noteworthy discrepancies in the total sound pressure distributions between P_1_ and M_z_, as shown in the rectangular dotted boxes of Fig. S6. Consequently, the transmission phase between P_1_ and M_z_ is anticipated to exhibit divergence as well.

Text S7. Angle- and frequency-dependent transmission behavior of P_1_, P_2_, and M_z_

These extended simulations show that the transmittance of M_z_ and P_1_ remains identical across all angles (Fig. S7A to C). The transmission phase, however, exhibits a mirror-symmetric angular response, resulting in phase mismatch compared to P_1_ for oblique incidence (Fig. S7D to F). In contrast, P_1_ and P_2_ show perfect agreement in both transmittance and transmission phase across the full angular range. The angular transmission phase difference between P_1_ and M_z_, plotted in Fig. S7G, quantifies the magnitude of this effect, confirming that parity transformation leads to the undistorted wavefront, rather than mirror transformation.

Text S8. Simulation of rugged terrain using parity metamaterial

In Fig. S8, we demonstrate a “rugged terrain”, which leads to randomly varying reflection phases (Fig. S8A). We simulated its 2D far-field radiation pattern under normal incidence (Fig. S8B), which shows a diffuse-like scattering signature. We then designed a parity metamaterial configuration to emulate this phase profile using appropriate arrangements of P_1_ and P_2_ elements. The resulting structure and its simulated far-field reflection are shown in Fig. S8C and D, respectively.

Notably, the reflected energy distribution from the parity metamaterial closely resembles that of the target terrain (Fig. S8B vs. S8D), apart from an expected amplitude reduction due to transmission. More importantly, the transmitted wavefront remains unchanged, which ensures that a sonar system placed behind the metamaterial can function without distortion. This dual behavior—tunable reflection combined with undistorted transmission—shows the advantage of our design.

Text S9. Multiple-beam reflection with undistorted transmission

The diffraction grating equation describes the angles at which acoustic waves of a particular wavelength will be diffracted when they pass through a diffraction grating. The equation is:

$\sin\theta_{m}=\frac{m}{D}\lambda$ (S8)

Where $\theta_{m}$ is the diffraction angle, $\lambda$ is the wavelength of the incident wave, D is the length of the supercell ($D=2A=12 cm$ in our case), and m is the diffraction order (an integer). In Eq. S8, to fulfill the requirement $\left| sin\theta_{m} \right|\leq1$, m should be taken as $0, \pm1$ ($\lambda=6.04 cm$). When $m=0$, a phase difference of π between the meta-atom and its parity-inverted counterpart leads to vanishing reflection. Consequently, the reflection by the parity metamaterial has only two identical beams ($m=\pm1$), as shown in Fig. 4D.

It is noted that the reflection phase difference between P_1_ and P_2_ varies significantly with the rotation angle, covering -180° to 180°. Next, we demonstrate the simultaneous existence of three-beam reflection and undistorted transmission for the rotation angle of $\theta=42^{\circ}$. The metamaterial design is shown in Fig. S10A. At this time, the reflection phase difference between P_1_ and P_2_ becomes 90°. Therefore, the acoustic path difference is ${A sin\theta}_{m}+\frac{\lambda}{4}$. From Eq. S8, the acoustic path difference between P_1_ and P*_2_* within a unit cell is $\frac{(2m+1)\lambda}{4}$. When $m=0, \pm1$, the phase difference between P_1_ and P*_2_* are $\pm\frac{\lambda}{4}$ and $\frac{3\lambda}{4}.$ Therefore, the three beams can coexist on the side of reflection. Figure S10B shows the simulated 3D far-field radiation power pattern under normal incidence at 5.68 kHz when $\theta=42^{\circ}$. The phenomena of undistorted transmission and three-beam reflection can be observed simultaneously.

Text S10. Quantitative analysis of the transmission and reflection behaviors

To quantitatively evaluate the transmission consistency and the switching of reflection behavior, we have performed a detailed phase analysis of the acoustic field distributions shown in Fig. 4. Specifically, we extracted the acoustic phase profiles along a series of line segments located 15 cm or 25 cm away from both the parity metamaterial and a reference metamaterial composed entirely of P_1_-type units. These lines are numbered (1-6) on the transmission side and (7-9) on the reflection side, as shown in Fig. S11. Fig. S11A and B shows that the parity metamaterial achieves undistorted transmission while switching the reflection behavior from two-beam reflection to specular reflection. Fig. S11C shows the reference metamaterial case, which behaves as a homogeneous structure and yields specular reflection and undistorted transmission, as expected. We then plotted the phase data along the selected lines:

Fig. S11D demonstrates that the transmitted wavefront from the parity metamaterial matches that from the reference structure very well, confirming transmission consistency.

Fig. S11E shows distinct reflection behaviors: line 7 confirms the presence of two-beam reflection, whereas lines 8 and 9 both display specular profiles. Although the phase on line 8 is slightly delayed compared to line 9, the wavefront shape remains consistent, supporting the conclusion of specular reflection.

Text S11. Diffuse reflection with undistorted transmission

We demonstrate the case of diffuse reflection and undistorted transmission by designing a parity metamaterial with random configurations, as shown in Fig. S12A. When the rotation angle of P_1_ (P_2_) is $\theta=0^{\circ}$, the reflection phase difference between P_1_ and P_2_ is 180°. We plot the simulated 3D far-field radiation power pattern under normal incidence at 5.68 kHz, as shown in Fig. S12B. A prominent radiation lobe is clearly seen in the direction of incidence, which corresponds to the transmission (P_t_) through the parity metamaterial. However, the reflection lobes (P_r_) of the parity metamaterial spread in many different directions. For clarity, the far-field reflection pattern in a logarithmic coordinate is also shown in the inset of Fig. S12B. It is clearly seen that the reflected-wave energy is distributed uniformly, which is the characteristic feature of diffuse reflection. Diffuse reflection is also one of the most desired functions for a sonar dome, besides the basic requirement of ultra-broadband undistorted transmitted wavefront for detection.

Text S12. Reflection holography with undistorted transmission

Here, we also demonstrate the case of reflection holography and undistorted transmission by the parity metamaterial. To enhance imaging accuracy, we design a larger parity metamaterial with $30\times30$ units. The metamaterial design for holography is shown in Fig. S13A, which is obtained by the angular spectrum method. The target image is shown in Fig. S13B, with the target focal length set to $f_{d}=20A=1.2 m$. In Fig. S13D, we plot the simulated near-field distribution of the transmitted acoustic field under normal incidence at 5.68 kHz. Clearly, the direction of transmission (*P_t_*) is the same as that of the incidence (*P_i_*). In Fig. S13E, we plot the simulated intensity profile of the reflected wave, where the letter 'U' is clearly visible. We emphasize that the imaging accuracy can be further improved by increasing the size of the parity metamaterial.

Text S13. Transmission distortion in conventional digital coding metasurface

Conventional digital coding metasurfaces can effectively manipulate reflected wavefronts and have been widely used to realize acoustic camouflage and reflection control functionalities. However, our work fundamentally distinguishes with them by achieving reflection control while preserving broadband undistorted transmission—a functionality beyond conventional digital metasurfaces.

In most reflection-type digital metasurfaces, meta-atoms are designed to produce distinct reflection responses, with little or no consideration for transmission. Even when partial transmission occurs, the associated wavefront is usually changed by the inhomogeneous digital metasurfaces, leading to distortion in the transmitted field. In contrast, our parity metamaterials are constructed from a pair of meta-atoms related by parity transformation, which—by reciprocity—guarantee identical transmittance and transmission phase, enabling broadband undistorted transmission. This unique combination enables sonar domes that simultaneously allow sonar detection and camouflage in reflection, which are of particular interest in stealth applications.

To illustrate this difference more clearly, we refer to a representative example from the literature: Appl. Phys. Lett. 119, 253903 (2021) [50], where a metasurface composed of multiple digital meta-atoms was used to control reflection [see Fig. S14A]. We arbitrarily selected two of their meta-atoms (labels 2 and 5, red box), and modified them by adding a bottom opening (radius $r=22 mm$), such that both reflection and transmission could occur—structure shown in Fig. S14B.

We computed the transmission characteristics of these modified meta-atoms. As shown in Fig. S14C, their transmittance and transmission phase differ significantly across 1–3.3 kHz, indicating that a metasurface constructed using such units would distort the transmitted wavefront.

We constructed a 1D metasurface from these two units [Fig. S14D], and simulated its near-field and far-field acoustic field. As shown in Fig. S14E to F, the transmitted field splits into three beams, completely destroying the original wavefront—a clear failure to maintain undistorted transmission.

In contrast, parity metamaterials constructed from reciprocal, parity-related unit pairs, maintain the same transmission behavior across both unit types, as validated in Fig. 2A and B. In other words, parity metamaterials are equivalent to homogeneous media in transmission, but equivalent to inhomogeneous media in reflection. Such a distinct property has not been observed or discussed in other acoustic materials before.

Text S14. The mechanism of the acoustic camouflage

We consider a scenario where a parity metamaterial is positioned at a distance d from a sonar. When an incident wave interacts with the parity metamaterial, a portion of the wave is reflected (characterized by the reflection coefficient r_1_), while the remaining part propagates through the parity metamaterial (described by the transmission coefficient t_1_), as shown in Fig. S15. As the transmitted wave traverses the air layer, it undergoes a phase shift, resulting in a transmission coefficient of $t_{1}e^{-i\varphi_{d}}$, where $\varphi_{d}$ represents the phase change through the air layer. The wave reflected back from the sonar is characterized by the coefficient $t_{1}{r_{2}e}^{-i\varphi_{d}}$, where r_2_ is the reflection coefficient of the sonar. Consequently, the wave returning to the parity metamaterial carries a coefficient of $t_{1}{r_{2}e}^{-2i\varphi_{d}}$. This wave is subsequently divided into two components: the reflected wave and the transmitted wave, with coefficients of $t_{1}r_{1}^{'}{r_{2}e}^{-2i\varphi_{d}}$ and $t_{1}^{2}{r_{2}e}^{-2i\varphi_{d}}$, respectively. It should be noted that $r_{1}^{'}$ denotes the reflection coefficient on the back interface of the parity metamaterial. These scattering cascades occur infinitely. The total reflection R can be expressed as

$R=r_{1}+\sum_{n=1}^{\infty} t_{1}^{2}{r_{1}^{'}}^{n-1}r_{2}^{n}e^{-2in\varphi_{d}}=r_{1}+t_{1}^{2}r_{2}e^{-2i\varphi_{d}}{(1-r_{1}^{'}r_{2}e^{-2i\varphi_{d}})}^{-1}$ (S9)

Here, we consider the case of normal incidence. By setting $R=0$, the overall specular signal can be eliminated. At this condition, we only need to consider the 0^th^-order transmission and reflection coefficients of the parity metamaterial and sonar, which can be computed using the commercial finite element software COMSOL Multiphysics. For example, when the rotation angle of the rotors is $\theta=75^{\circ}$, the calculated transmission and reflection coefficients are $t_{1}=-0.17569-0.20617i$, $r_{1}=-0.28323+0.434901i$, $r_{1}^{'}=-0.28334+0.43574i$, and $r_{2}=1.1289-0.11148i$, respectively. Substituting these values into simplified Eq. S9 yields a series of values for *d*. We have selected an appropriate value of $d=5.9 \mathrm{mm}$.

Actually, higher-order terms in reflection here are much smaller. Particularly, we compared the reflection cancellation point calculated under two scenarios: (i) a full multiple-reflection model (Fig. S15), and (ii) a simplified model considering only the first and second reflections. The computed optimal distances between the parity metamaterial and the sonar system are 5.9 mm and 7.1 mm, respectively, indicating a small deviation.

Text S15. Meta-atom and its parity-inverted counterpart with loss

We emphasize that the design strategy presented in this work is universal and applicable to scenarios with loss. We take P_1_ and P_2_ from Fig. 1 in the main text as an example. Here, the dissipation term here is characterized by adding a 10% imaginary part in the bulk modulus of air. Figure S16A and B shows, respectively, the calculated transmittance and transmission phase, and the reflectance and reflection phase of P_1_ and P_2_, as functions of the frequency. Clearly, even in the presence of loss, P_1_ and P_2_ have identical transmittance and transmission phase over an ultra-broad spectrum ranging from 0.1 to 7 kHz. This characteristic ensures that the parity metamaterials composed of P_1_ and P_2_ can keep the transmission wavefront undistorted in the ultra-broad spectrum. On the other hand, P_1_ and P_2_ exhibit a slight difference in reflectance and a notable distinction in reflection phase, which reaches 180° at 5.78 kHz. Such a characteristic satisfies the requirement for designing dynamic reflection functionality. The simulated 3D far-field radiation power pattern and the corresponding near-field distributions in the yz-plane are calculated under normal incidence at 5.78 kHz, as shown in Fig. S16D and E, respectively. The combination of undistorted transmission and two-beam reflection is clearly observed. Therefore, our design strategy is universally applicable to scenarios with loss.

Text S16. Performance of the parity metamaterial underwater

Here, taking account of the acoustic-elastic coupling, we investigate the performance of the parity metamaterial underwater. We consider the parity metamaterial with a random distribution of P_1_ and P_2_ underwater, as shown in Fig. S17A. In underwater applications, the material for parity metamaterial can be chosen to be steel. The parameters of steel are taken to be mass density $\rho=7850 kg/m^{3}$, the Young's modulus $E=180 Gpa$, and Poisson ratio $\upsilon=0.25$. The parameters of water are set as $\rho_{0}=1000 kg/m^{3}$ and $c_{0}=1531 m/s$. Fig. S17A and B illustrates, respectively, the calculated transmittance and transmission phase, and the reflectance and reflection phase of P_1_ and P_2_, as functions of the frequency. Clearly, even underwater, P_1_ and P_2_ have identical transmittance and transmission phase over an ultra-broad spectrum ranging from 26 to 32 kHz. This characteristic ensures that the parity metamaterials composed of P_1_ and P_2_ can manifest undistorted transmission wavefront. On the other hand, P_1_ and P_2_ exhibit slight differences in reflectance, but there is a notable distinction in the reflection phase. The reflection phase difference between P_1_ and P_2_ can reach 180° at 28.95 kHz. Such a characteristic satisfies the requirement for designing dynamic reflection functionality. Fig. S17D and E shows the simulated 3D far-field radiation power pattern and corresponding near-field distribution in the yz-plane under normal incidence. The phenomena of undistorted transmission and diffuse reflection are clearly observed. Therefore, our design strategy is applicable underwater.

Text S17. Parity metamaterials with richer degrees of freedom

Interestingly, when the thickness increases, more degrees of freedom could be introduced into the parity metamaterials, thereby enabling more ways of regulation. Here, we demonstrate a meta-atom with four different inclusions, i.e., a sphere, two square panels, and an oblique circular panel (angle *θ_1_*), distributed vertically, denoted as N_1_. Its parity-inverted counterpart, N_2_, is obtained by applying parity transformation to N_1_, as depicted in Fig. S18A. The adjacent meta-atoms are also separated by hard boundaries. The length and thickness of the meta-atom (its parity-inverted counterpart) are specified as $a=30 mm$ and $H=140 mm$, respectively. The square panels have cross sections of $18\times18$ cm^2^. The distances are $L_{1}=68 mm$, $L_{2}=15 mm$, and $L_{3}=120 mm$ (the distance between the sphere and the circular panel). The centers of the sphere and the circular panel, as well as the center of the meta-atom, lie on the same straight line. The edge of the sphere is aligned with the upper surface of the meta-atom. The radius of the sphere and cylinder is $r_{1}=10 mm$ and $r_{2}=12 mm$, respectively. The length and width of the two middle square panels are both $\omega_{1}=\omega_{2}=18 mm$. The thickness of the hard walls, square panels, and circular panel are all $t=2 mm$. The insets of Fig. S18A show the top view of the square panel and the front view of the circular panel, respectively. The angle between the circular panel and the *y*-axis is $\theta_{1}=45^{\circ}$*.* We plot the calculated transmission phase difference Δ*φ_t_* and reflection phase difference Δ*φ_r_* as functions of the incident angle and frequency in Fig. S18B and C. It is seen that Δ*φ_t_* is zero over an exceptionally broad spectrum spanning from 0.1 to 6 kHz and across wide angles from 0° to 50°. Conversely, due to the larger thickness, Δ*φ_r_* undergoes multiple transitions from 0° to 360°. At 4.393 kHz, the reflection phase difference precisely reaches 180° (denoted by a black star in Fig. S18C).

Next, we demonstrate that this parity metamaterial of greater thickness exhibits richer degrees of freedom to modulate the reflection. The degrees of freedom we considered are the distances *L_1_*, *L_2_*, *L_3_*, and the angle of the oblique circular panel *θ_1_*. In Fig. S18D, we plot the calculated Δ*φ_t_* and Δ*φ_r_* as functions of *L_1_* and *L_2_* under the normal incidence at 4.393 kHz. It is observed that $\varphi_{t}=0$ regardless of the parameters *L_1_* and *L_2_*. However, Δ*φ_r_* varies with *L_1_* and *L_2_*, covering the whole region from 0° to 360°. Interestingly, the region between the two square panels forms a resonant cavity of length *L_2_*, as shown in the inset in Fig. S18A (obtained at parameters marked by a black star in Fig. S18D). We note that $L_{1}+L_{2}$ has a maximum value. In Fig. S18E and F, we plot the calculated Δ*φ_t_* and Δ*φ_r_* as functions of *L_3_* and *θ_1_*. Similarly, $\Delta\varphi_{t}=0$ regardless of the parameters *L_1_* and *L_2_*. On the contrary, Δ*φ_r_* varies significantly by adjusting *L_3_* and *θ_1_*, covering the whole region from 0° to 360°. This case shows that our design strategy is universal and applicable to general acoustic metamaterials.

**Figures**


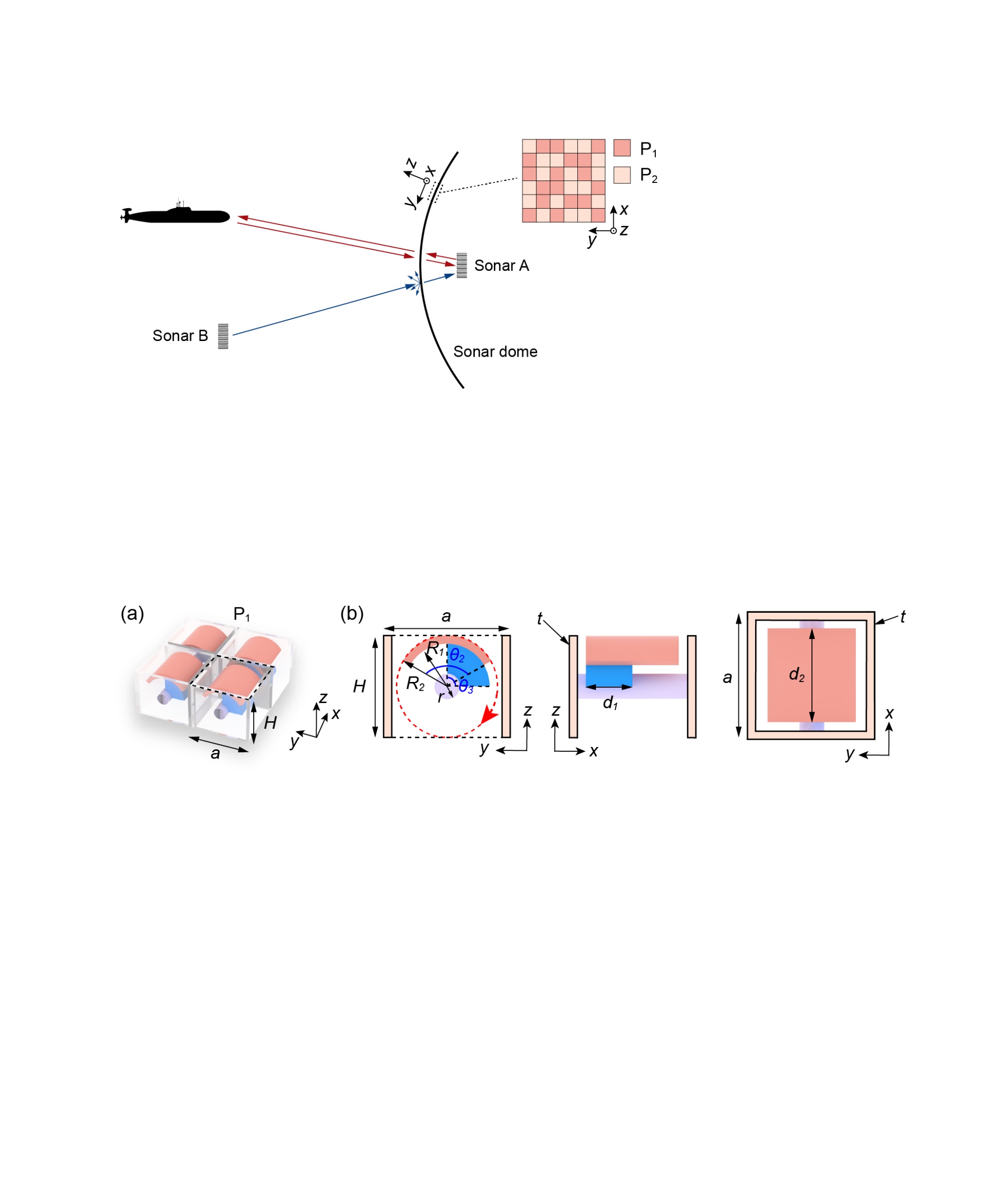


**Fig. S1.** Schematic of the camouflaged sonar dome based on parity metamaterials.

**
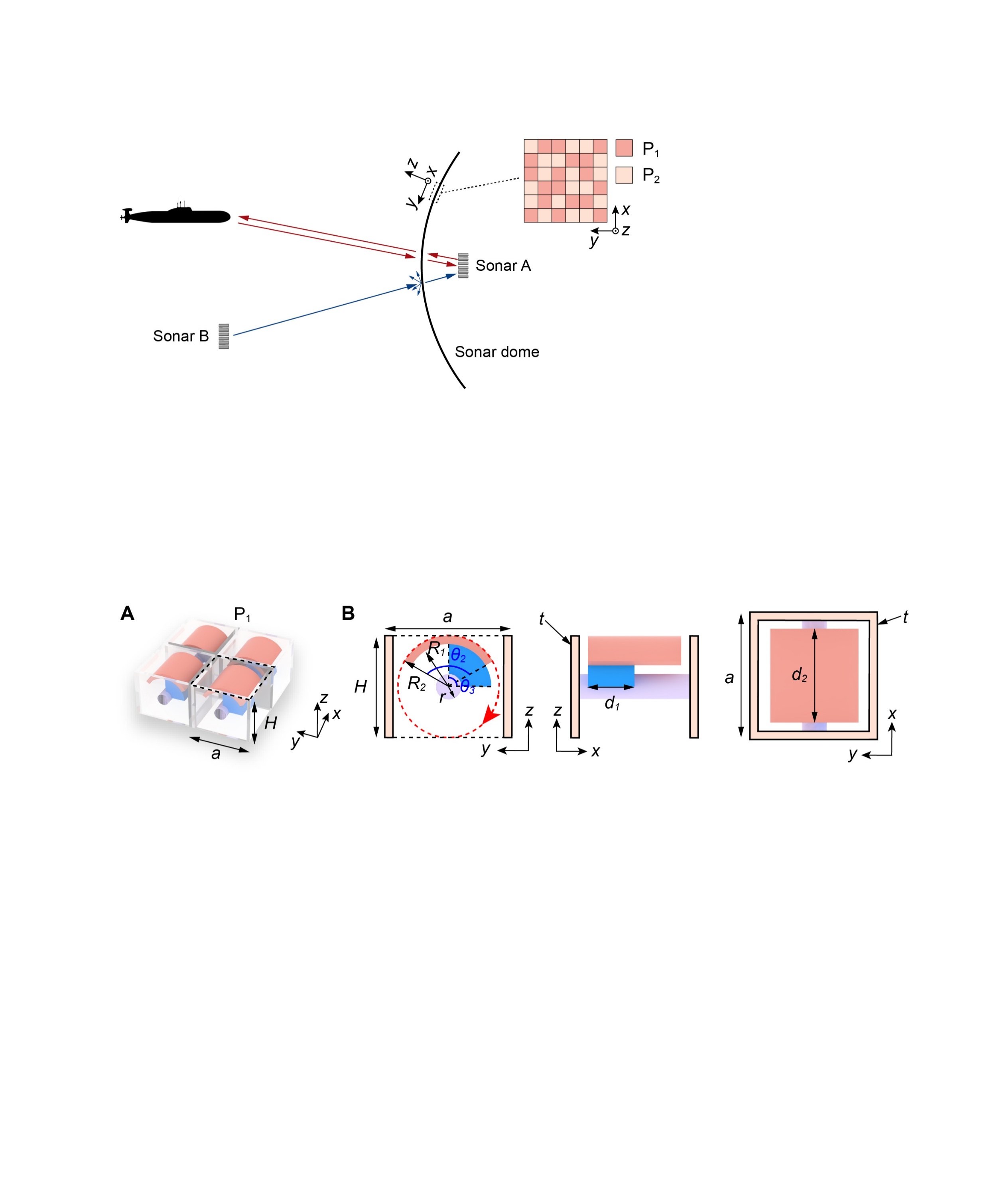
**

**Fig. S2.** Detailed parameters of the meta-atom. (A) Two-dimensional illustration of P_1_. (B) Front view, right view, and top view of the meta-atom.


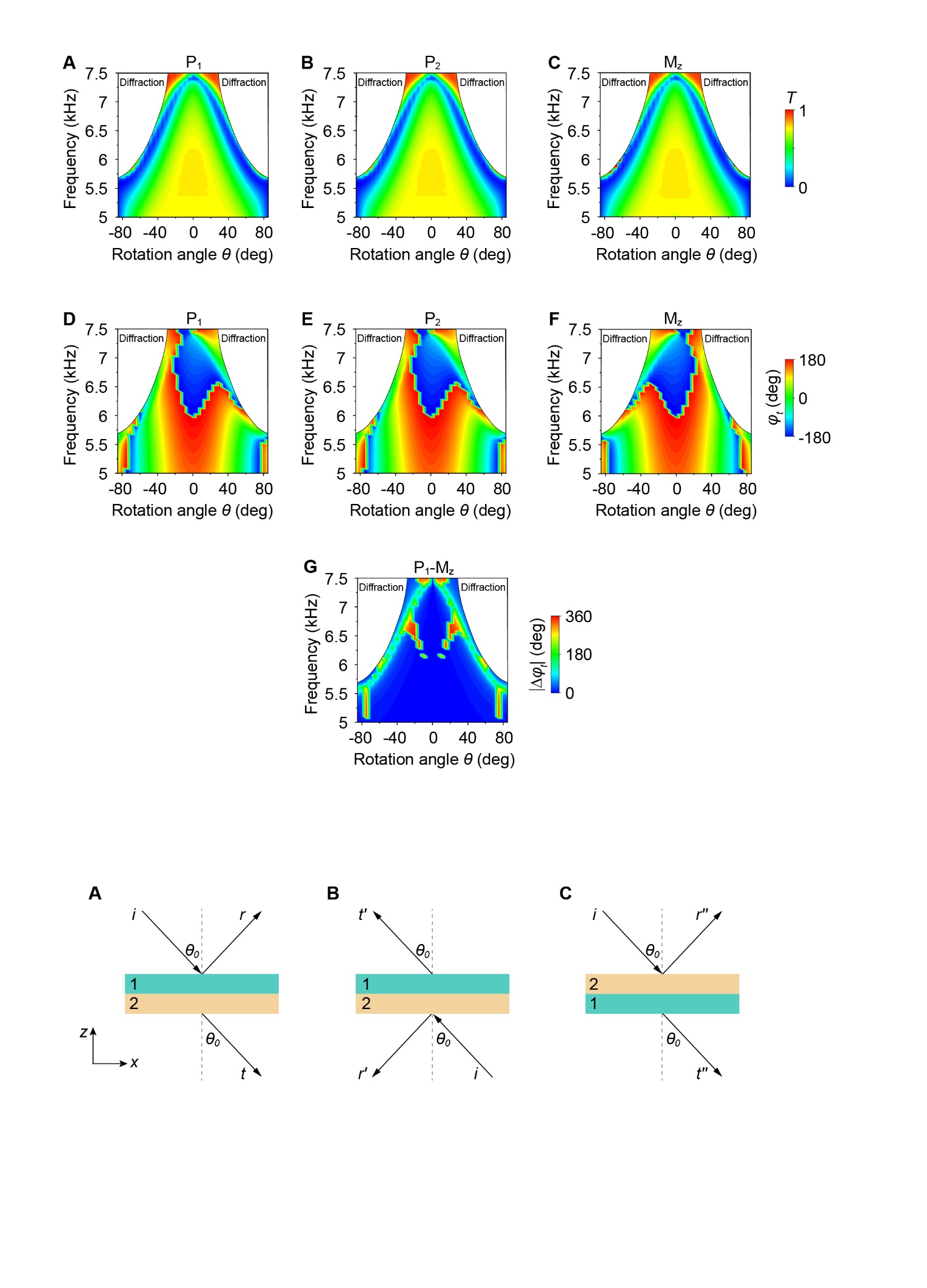


**Fig. S3.** Reciprocity-protected transmission of a stack and its parity-inverted counterpart.


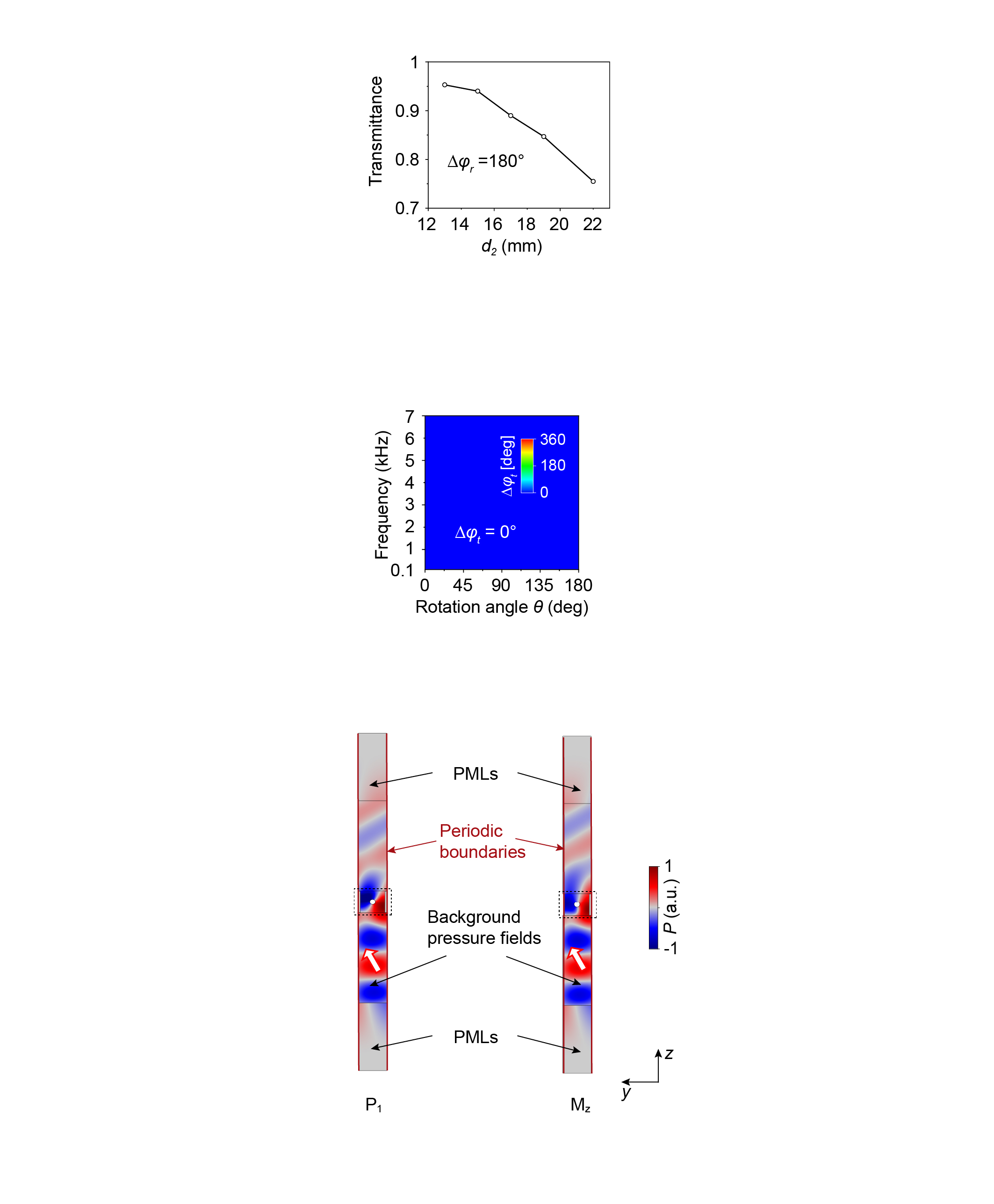


**Fig. S4.** Transmittance of P_1_ (P_2_) at the condition of reflection phase difference, $\Delta\varphi_{r}=180^{\circ}$, as a function of the depth of the curved plate, *d_2_*.

**
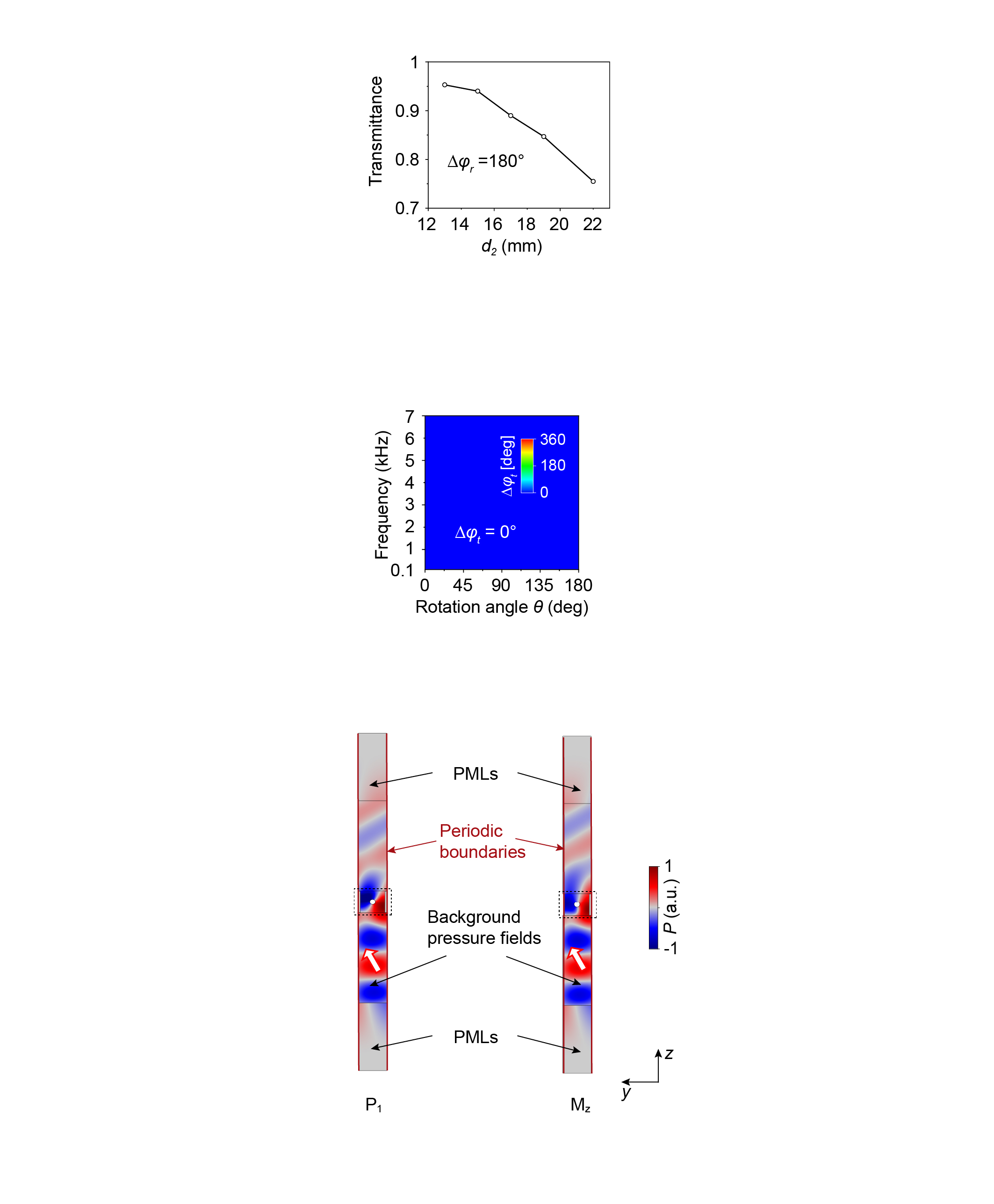
**

**Fig. S5.** Transmission phase difference between P_1_ and P_2_ as a function of the rotation angle of the rotor and frequency.


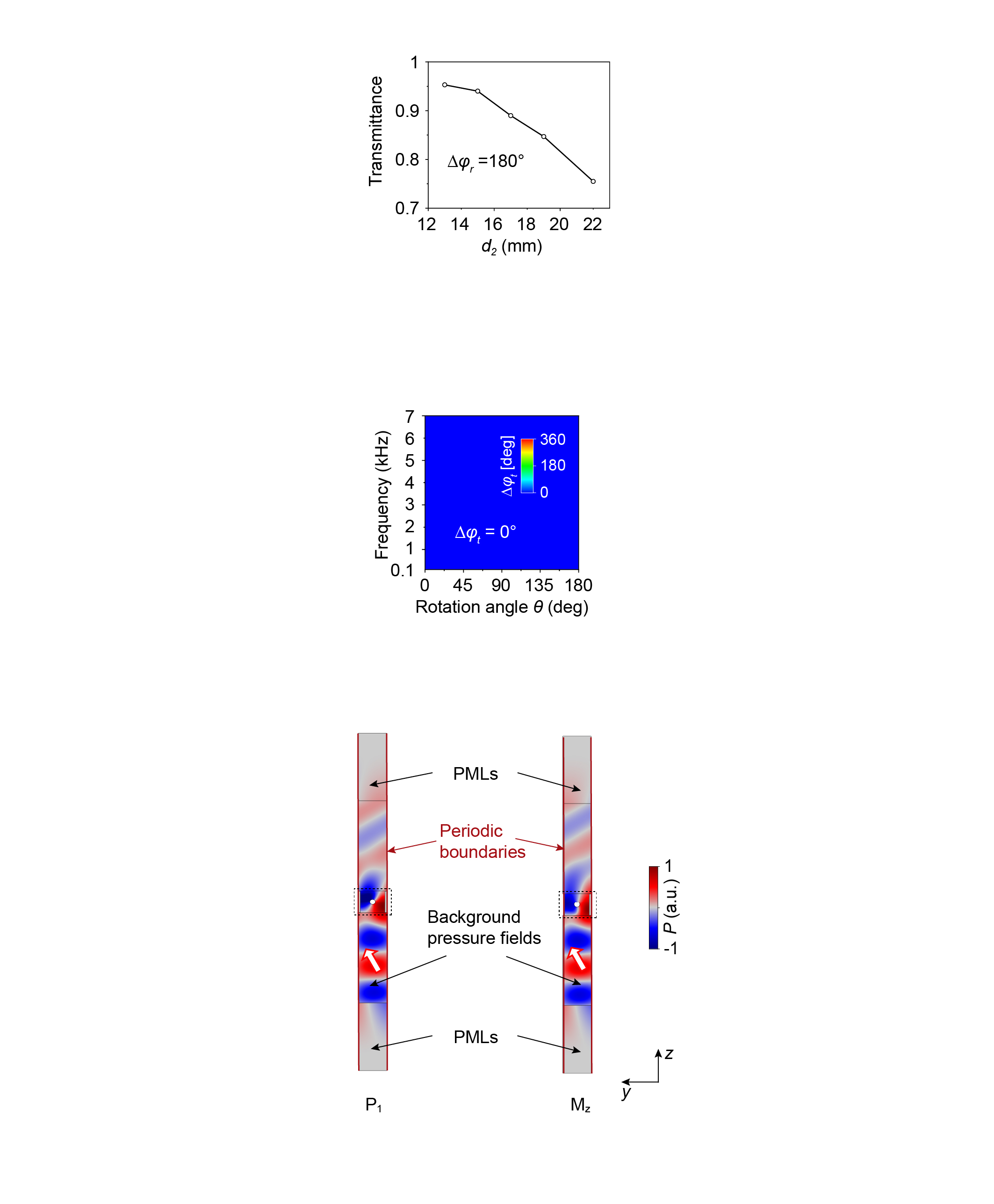


**Fig. S6.** Simulated sound pressure distributions of P_1_ and M_z_.


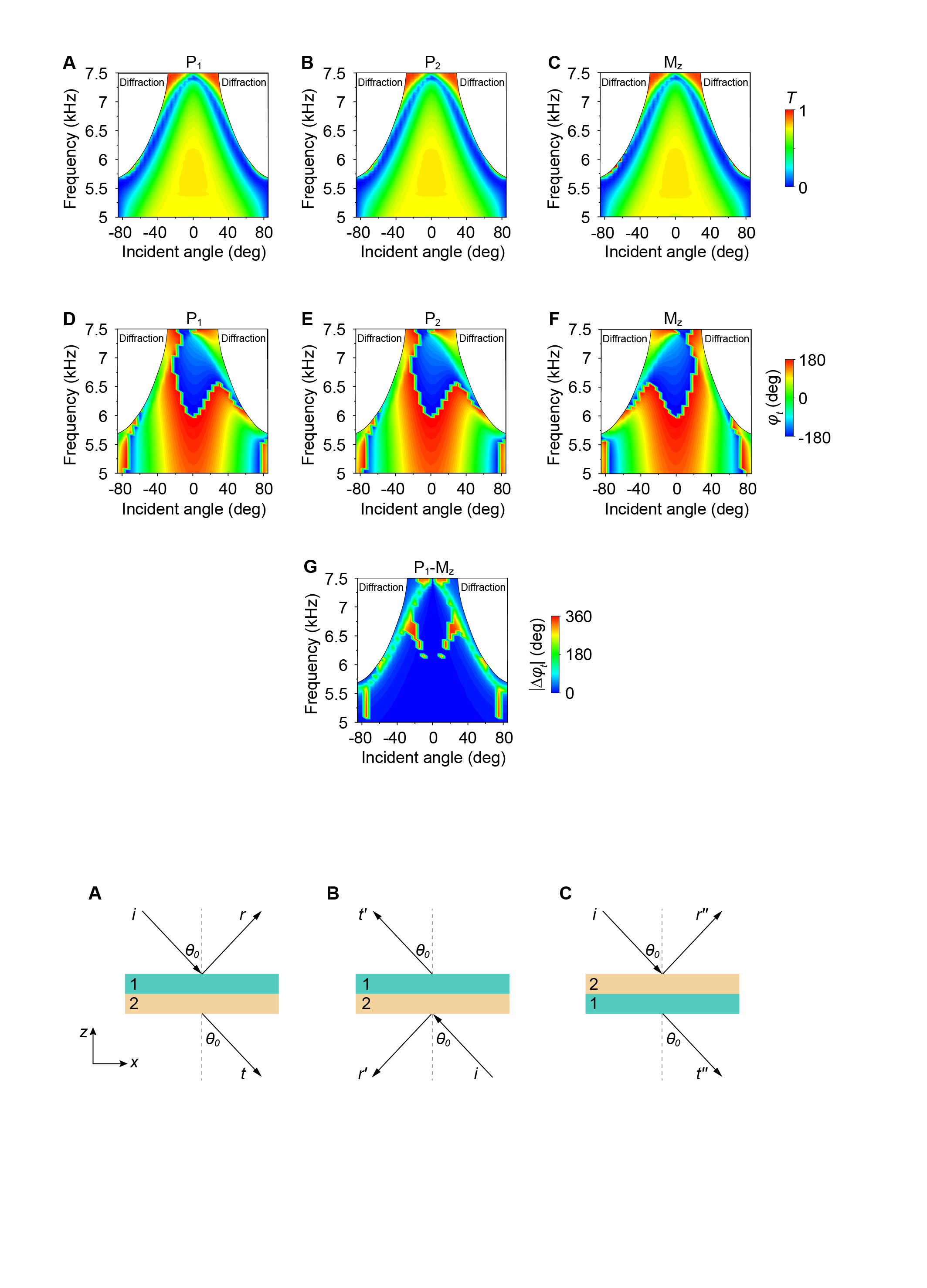


**Fig. S7.** Angle- and frequency-dependent transmission behavior of P_1_, P_2_, and M_z_ structures. (A to C) Transmission amplitude of P_1_, P_2_, and M_z_ as functions of incidence angle and frequency. (D to F) Transmission phase of P_1_, P_2_, and M_z_ as functions of incidence angle and frequency. (G) Transmission phase difference between P_1_ and M_z_ across angle and frequency, highlighting their mismatch under oblique incidence.


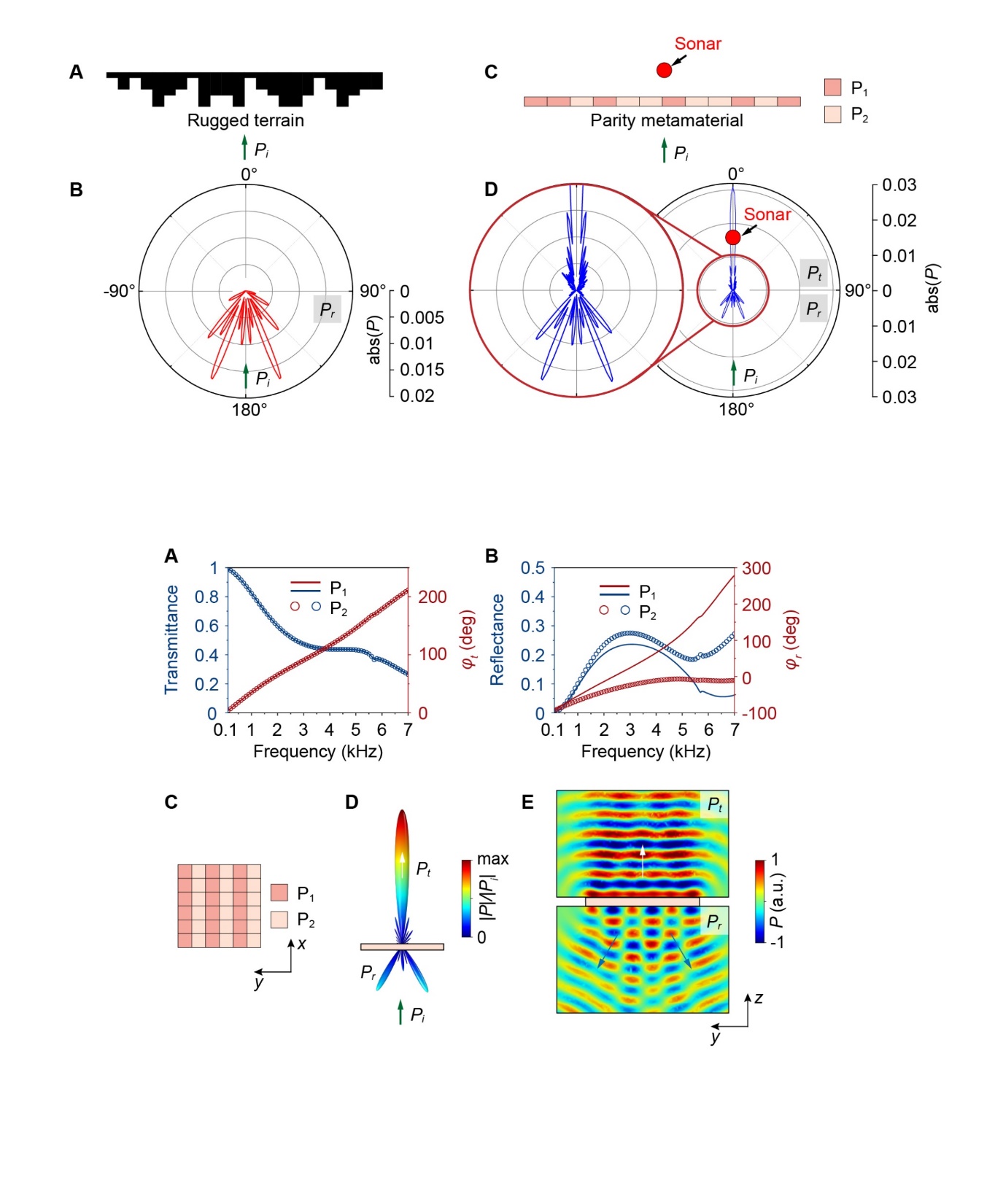


**Fig. S8.** Simulation of rugged terrain using parity metamaterial. (A and B) Simplified rugged terrain and its simulated 2D far-field radiation power pattern. (C and D) Design of a one-dimensional parity metamaterial and its corresponding simulated 2D far-field radiation power pattern.

**
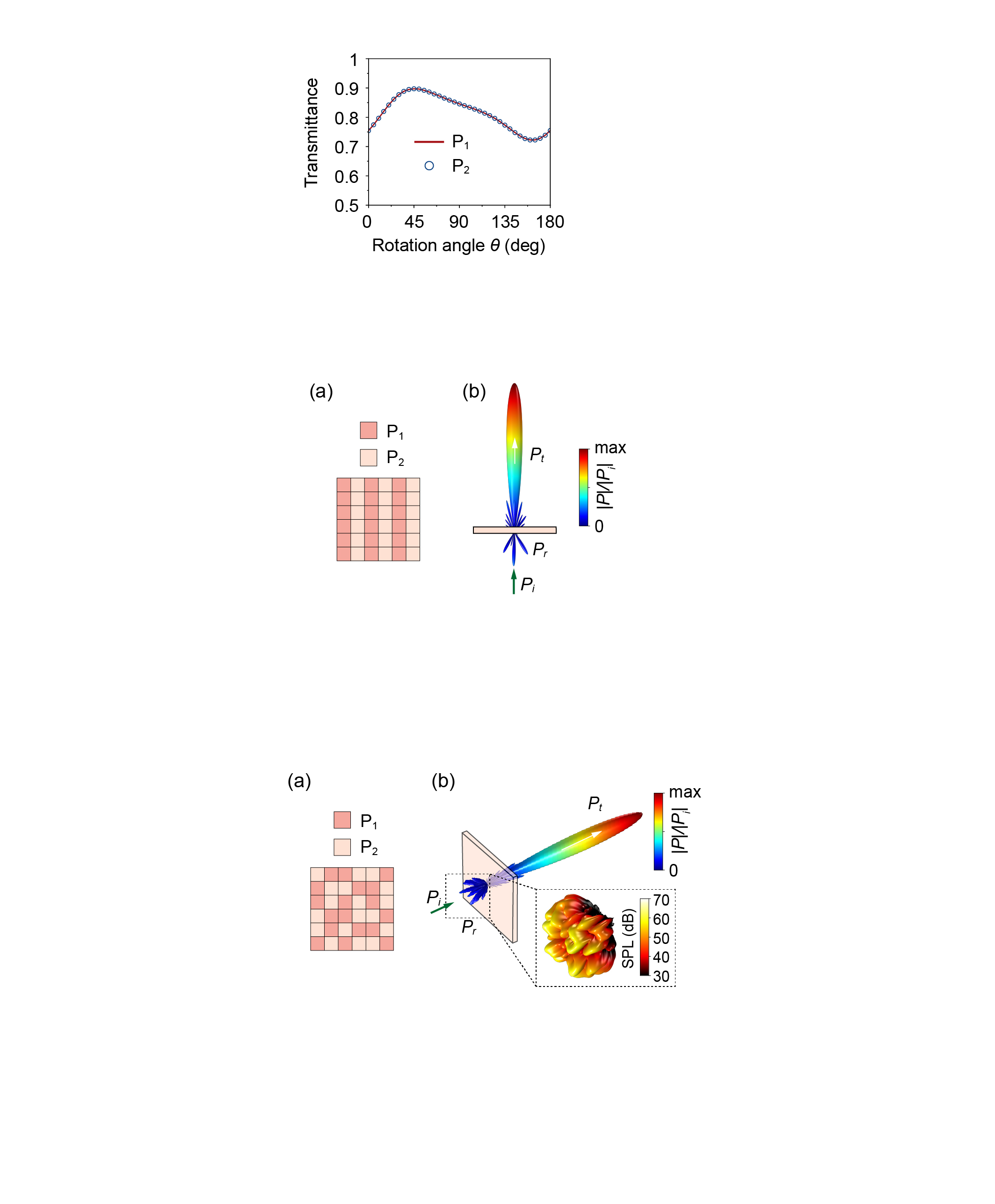
**

**Fig. S9.** Calculated transmittance of P_1_ and P_2_ regarding the rotation angle of the inner rotors at 5.68 kHz. P_1_ and P_2_ have identical transmittance due to reciprocity and parity transformation.

*
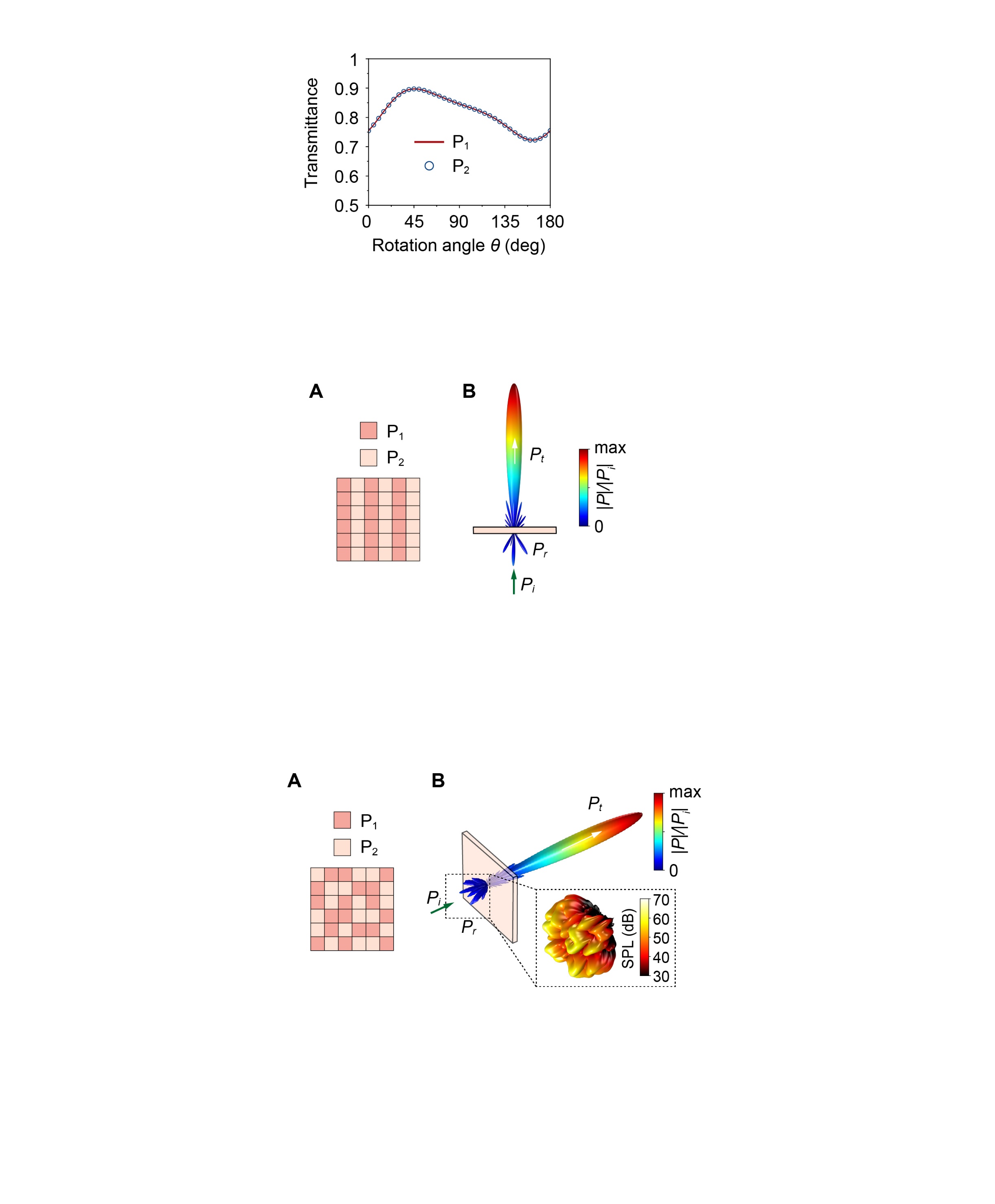
*

**Fig. S10.** Three-beam reflection with undistorted transmission. (A) The metamaterial design for three-beam reflection. (B) Simulated 3D far-field radiation power pattern under normal incidence at 5.68 kHz when $\theta=42^{\circ}$.


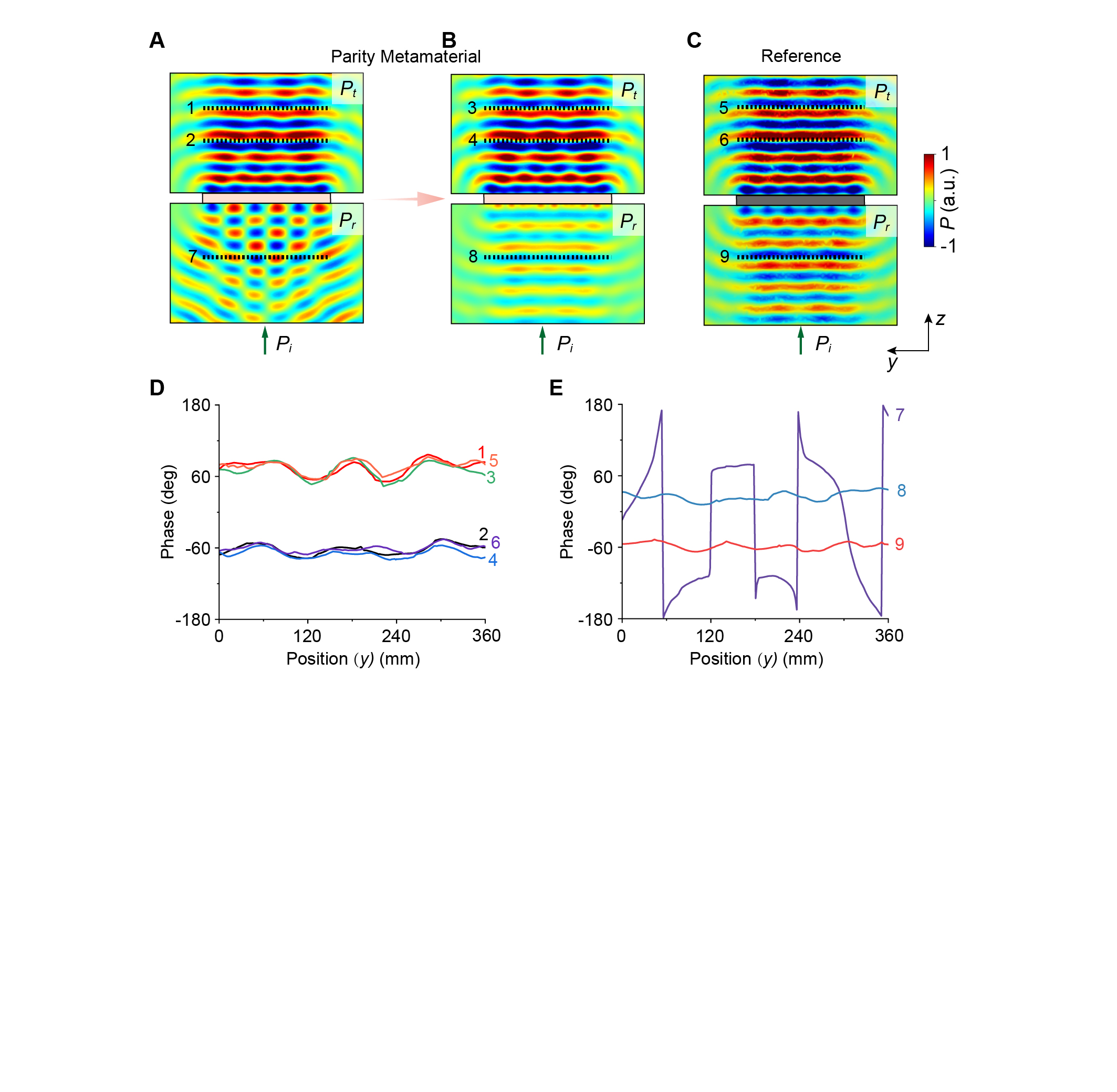


**Fig. S11.** Quantitative comparison of transmission and reflection phase profiles for the parity metamaterial and reference structure. (A) Simulated acoustic field distributions for the parity metamaterial, showing undistorted transmission and two-beam reflection. (B) Simulated acoustic field distributions for the parity metamaterial, showing undistorted transmission and specular reflection. (C) Simulated acoustic field distributions for a reference metamaterial composed entirely of P_1_ units, exhibiting undistorted transmission and specular reflection. (D) Extracted transmission phase profiles along lines 1-6. (E) Extracted reflection phase profiles along lines 7-9.

**
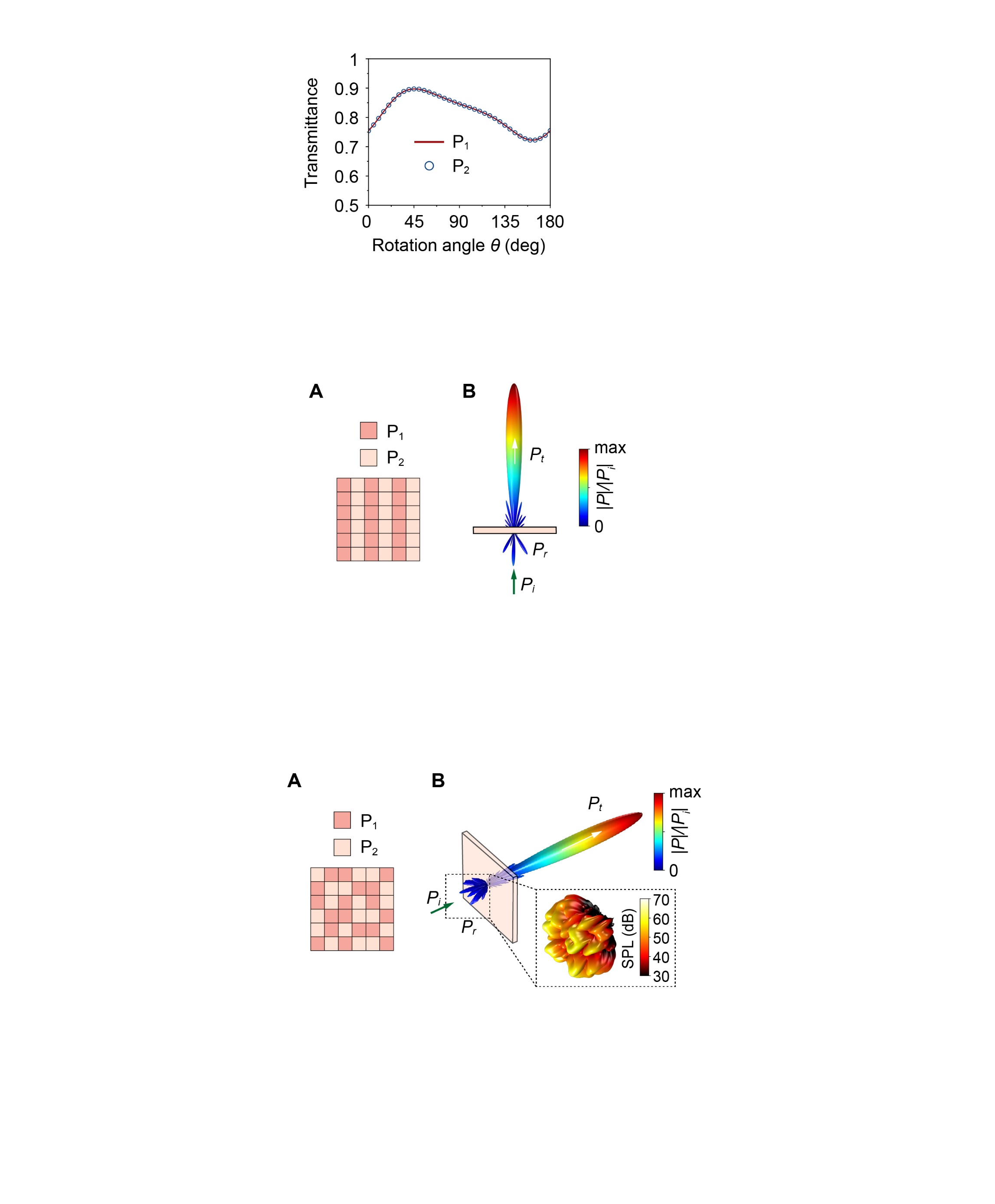
**

**Fig. S12.** Diffuse reflection with undistorted transmission. (A) The metamaterial design for diffuse reflection. (B) Simulated 3D far-field radiation power pattern under normal incidence at 5.68 kHz. The inset shows the far-field reflection patterns in a logarithmic coordinate.

**
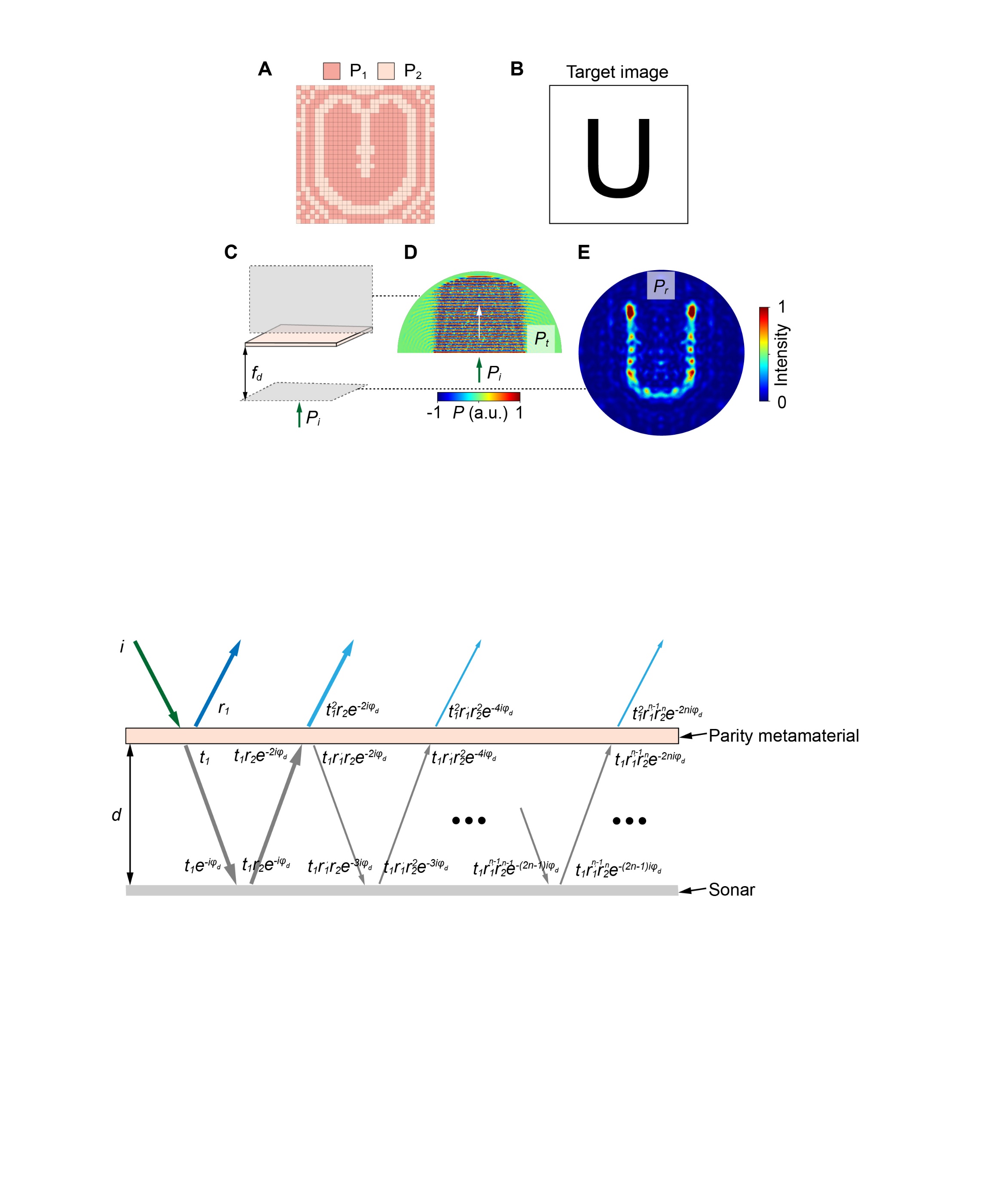
**

**Fig. S13.** Reflection holography with undistorted transmission. (A) The metamaterial design for holography. (B) Target image. (C to E) Simulated near-field distribution of the transmitted acoustic field (D) and the intensity profile of the reflected wave (E) under normal incidence at 5.68 kHz. The grey rectangular boxes correspond to the planes of the simulated near-field results.


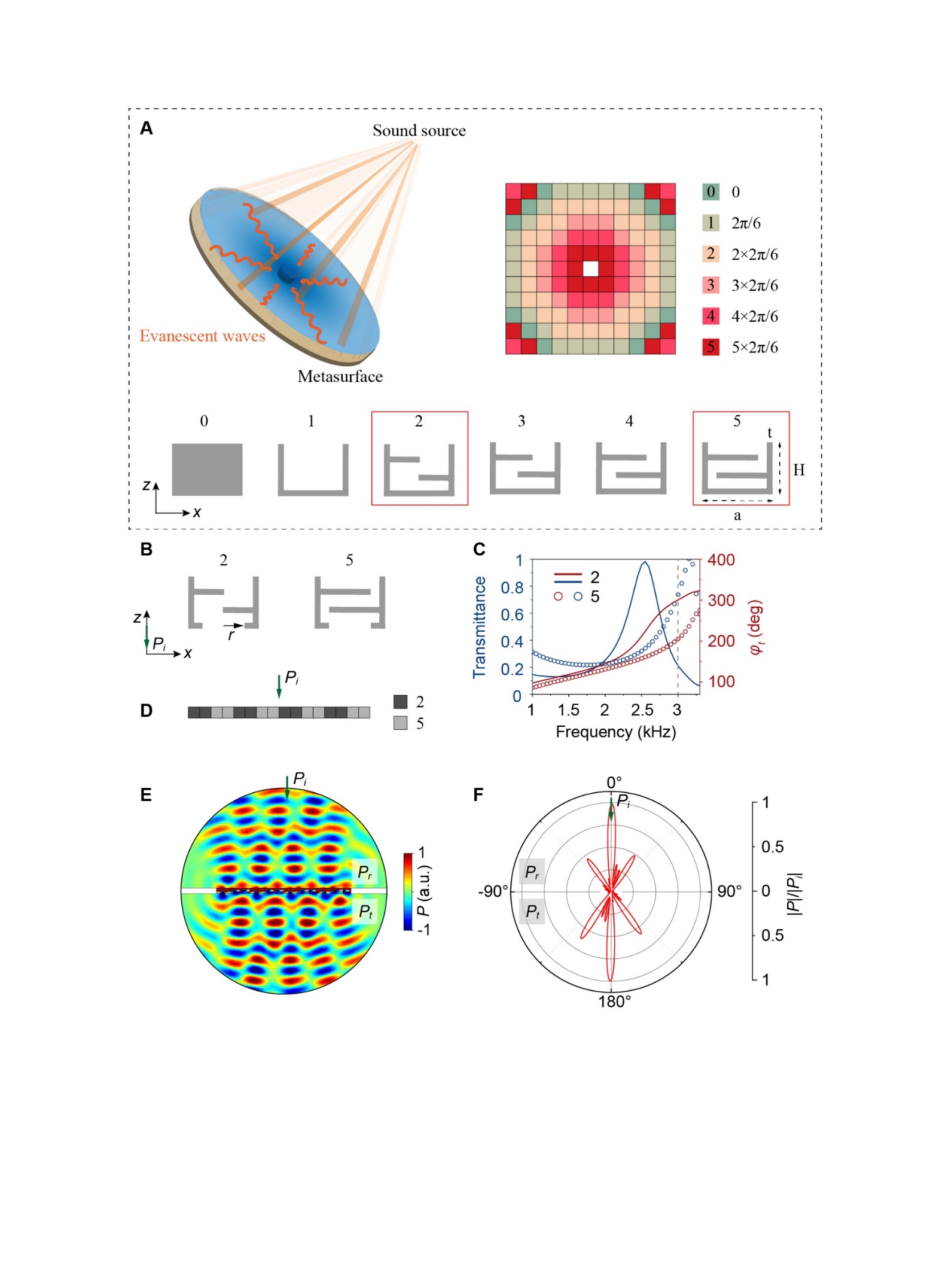


**Fig. S14.** Illustration of the mechanism of the acoustic camouflage.Transmission distortion in conventional digital coding metasurface. (A) A representative digital metasurface design from Appl. Phys. Lett. 119, 253903 (2021), composed of multiple meta-atoms with different reflection properties. Two meta-atoms (units 2 and 5, highlighted in red) are selected for comparison. (B) Modified versions of meta-atoms 2 and 5, with bottom openings (radius $r=22 mm$) added to allow both reflection and transmission. (C) Simulated transmittance and transmission phase of the two modified meta-atoms under normal incidence. (D) A 1D metasurface constructed using the two modified meta-atoms. The incident wave is marked by the green arrow. (E) Simulated near-field distributions of the reflected (upper) and transmitted (lower) acoustic fields of the 1D metasurface under normal incidence at 3.3 kHz, showing significant wavefront distortion in transmission. (F) Corresponding far-field radiation power pattern, revealing multiple transmitted beams and loss of wavefront integrity.

**
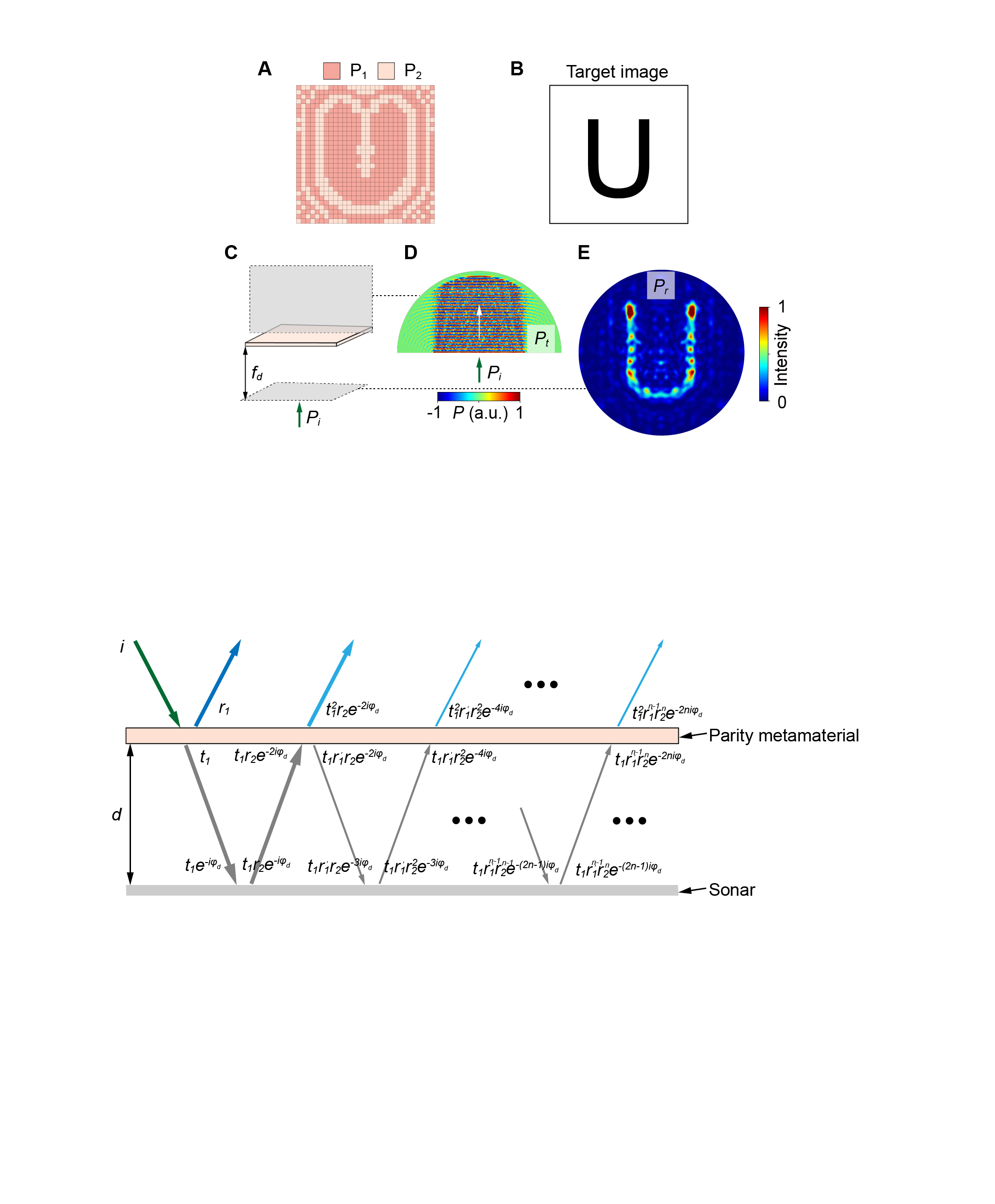
**

**Fig. S15.** Illustration of the mechanism of the acoustic camouflage.


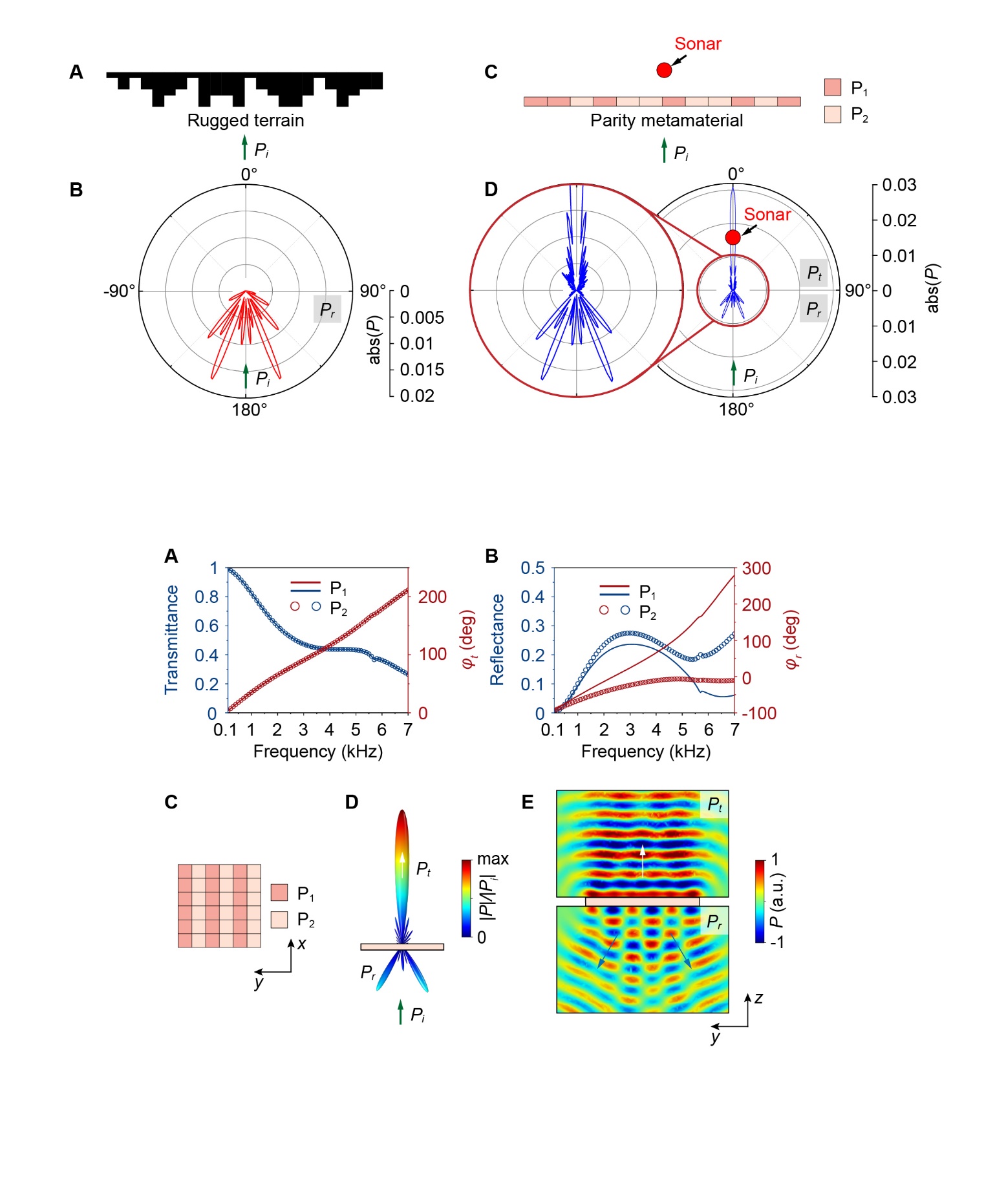


**Fig. S16.** Performance of the parity metamaterial with loss. (A) Transmittance and transmission phase spectra of P_1_ and P_2_. (B) Reflectance and reflection phase spectra of P_1_ and P_2_. (C) The metamaterial design. (D) Simulated 3D far-field radiation power pattern under normal incidence at 5.78 kHz. (E) Simulated corresponding near-field distributions in the *yz*-plane.

**
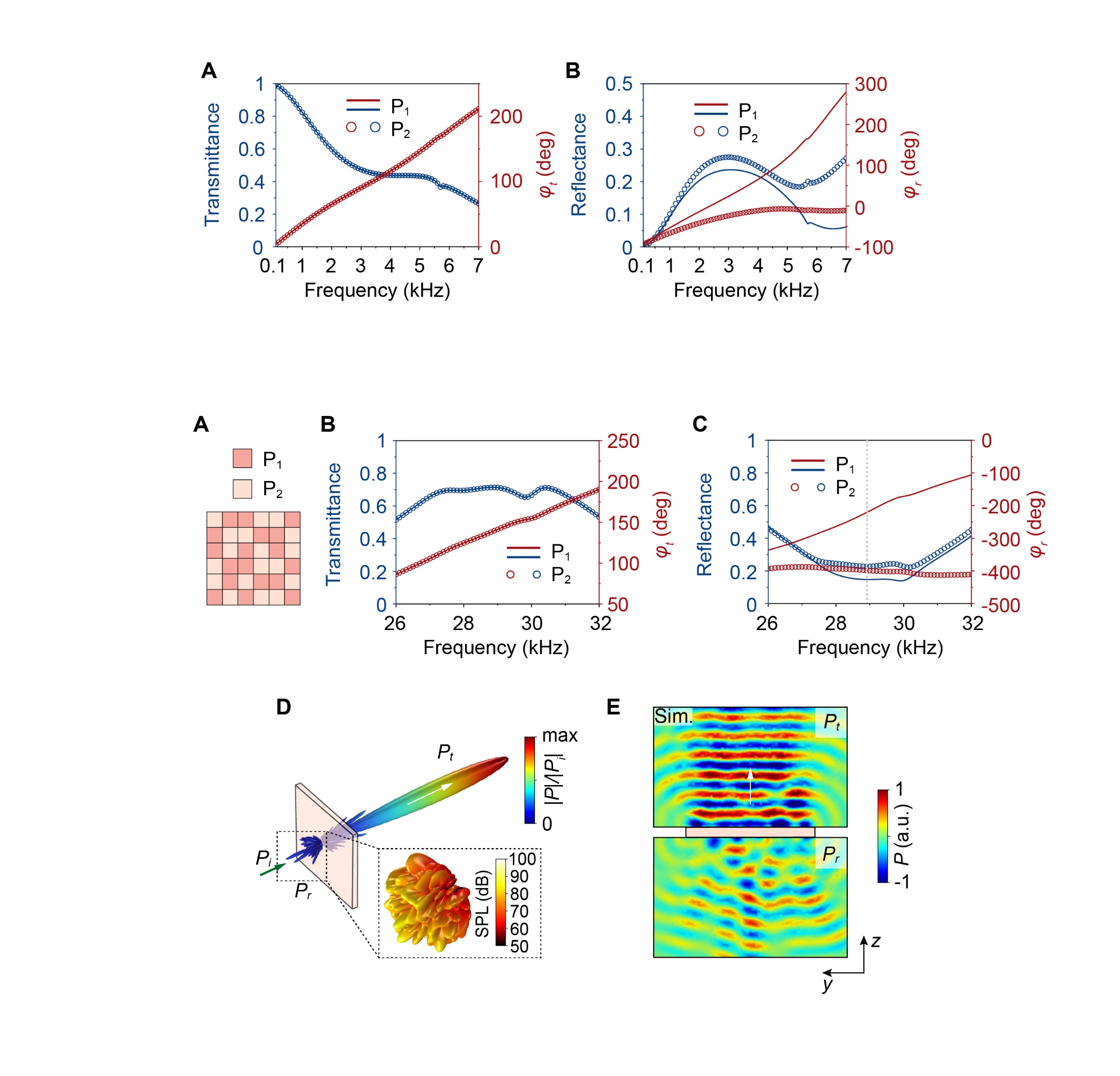
**

**Fig. S17.** Performance of the parity metamaterial underwater. (A) The design of the parity metamaterial underwater. (B) Transmittance and transmission phase spectra of P_1_ and P_2_. (C) Reflectance and reflection phase spectra of P_1_ and P_2_. (D) Simulated 3D far-field radiation power pattern under normal incidence at 28.95 kHz. (E) Simulated corresponding near-field distribution in the yz-plane.

**
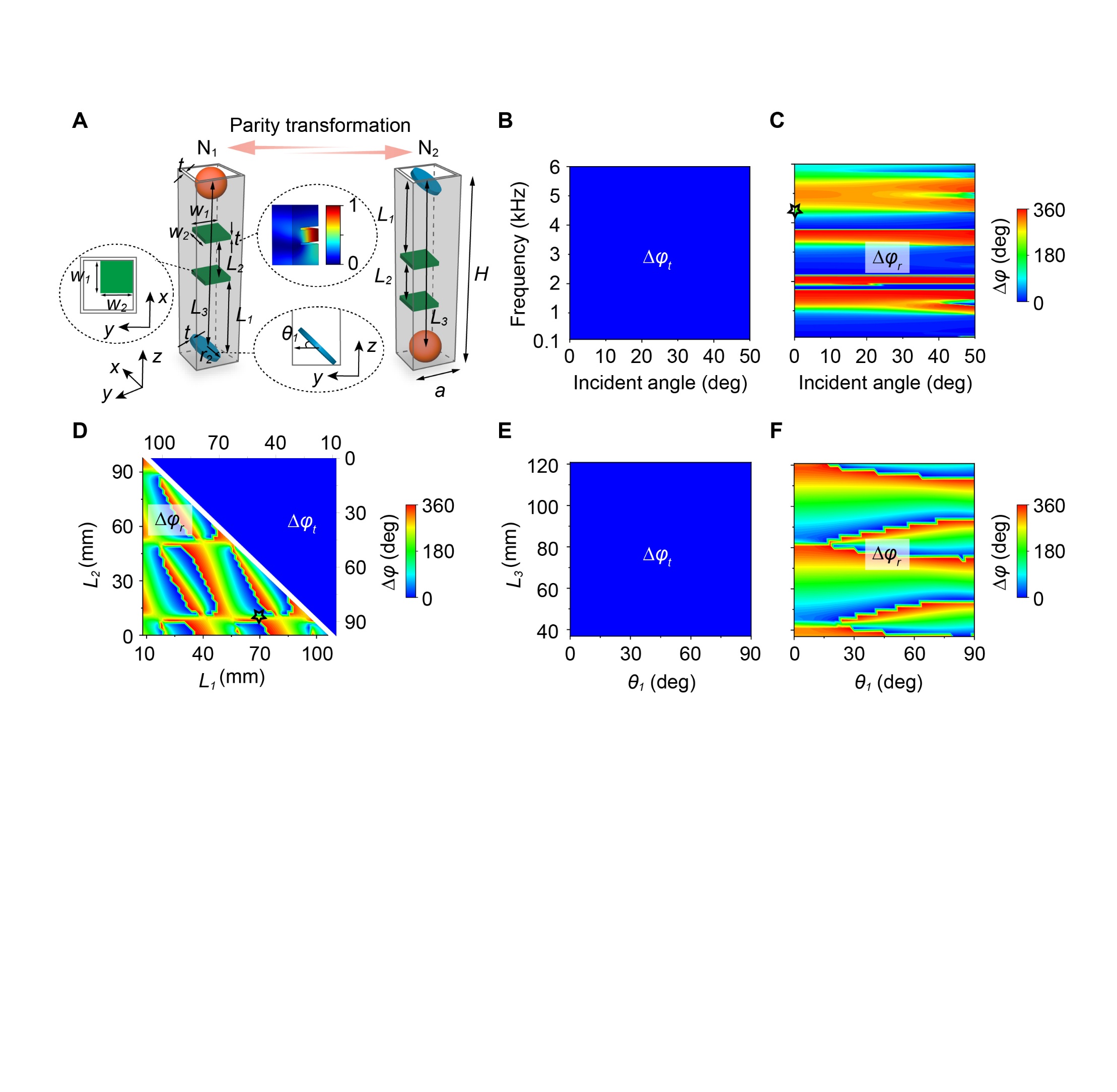
**

**Fig. S18.** Universality of the design strategy and more degrees of freedom. (A) Schematic diagrams of the meta-atom and its parity-inverted counterpart composed of four different inclusions. The upper inset displays a selected acoustic intensity profile of N_1_. The lower inset displays the side view of the circular panel. (B and C) Calculated transmission phase difference Δ*φ_t_* and reflection phase difference Δ*φ_r_* as functions of the incident angle and frequency. (D) Calculated transmission phase difference Δ*φ_t_* and reflection phase difference Δ*φ_r_* as functions of *L_1_* and *L_2_*. (E and F) Calculated transmission phase difference Δ*φ_t_* and reflection phase difference Δ*φ_r_* as functions of *θ_1_* and *L_3_* (with $L_{1}=22 mm$ and $L_{2}=10 mm$). The considered frequency is 4.393 kHz.

**
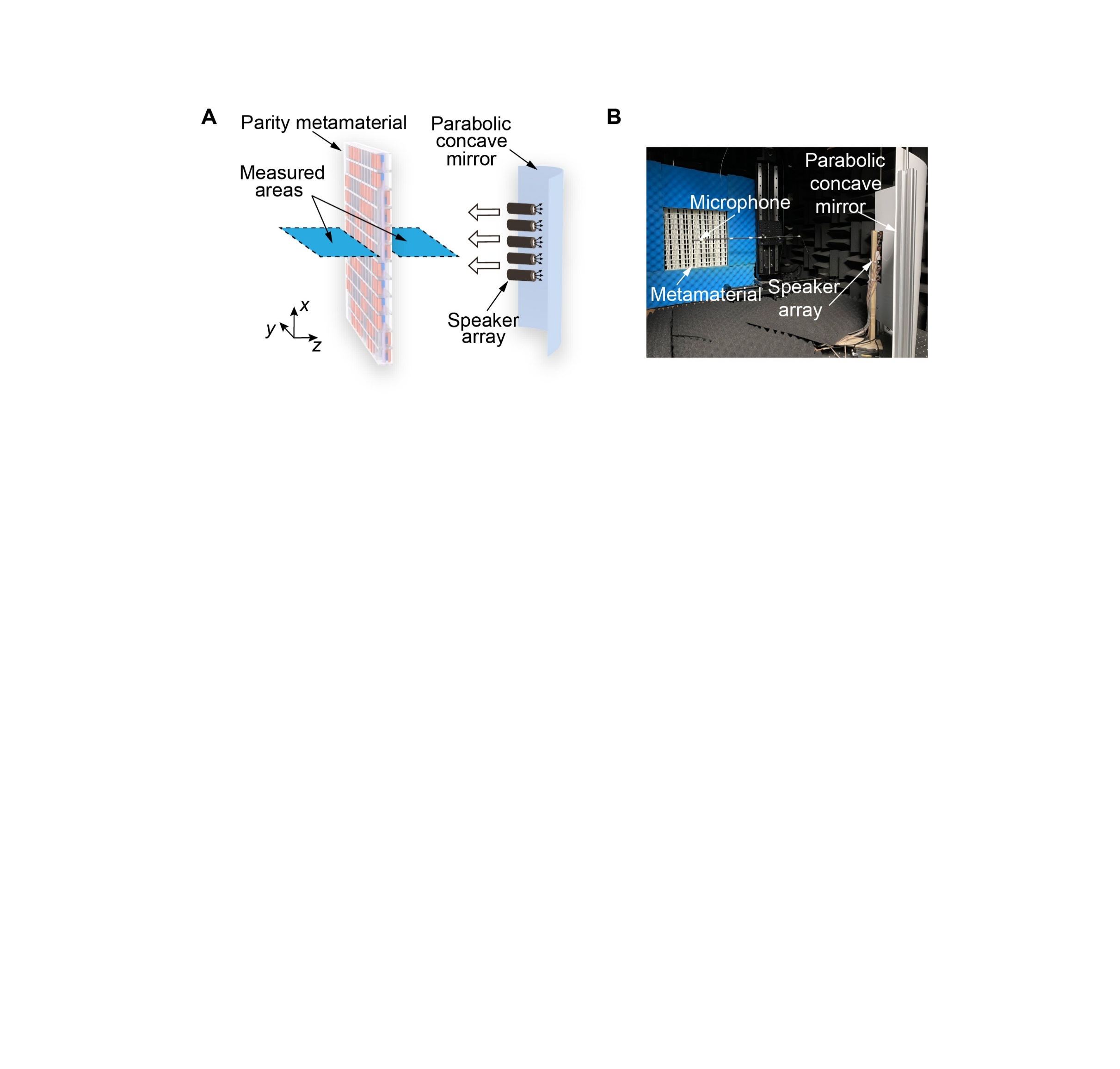
**

**Fig. S19.** Relevant experimental settings. (A) Schematic diagram of the experimental setup. (B) Photograph of the experimental setup.
